# Reduction of Cofed Carbon Dioxide Modifies the Local Coordination Environment of Zeolite-Supported, Atomically Dispersed Chromium to Promote Ethane Dehydrogenation

**DOI:** 10.1021/jacs.4c00995

**Published:** 2024-03-29

**Authors:** Wenqi Zhou, Noah Felvey, Jiawei Guo, Adam S. Hoffman, Simon R. Bare, Ambarish R. Kulkarni, Ron C. Runnebaum, Coleman X. Kronawitter

**Affiliations:** † Department of Chemical Engineering, University of California, Davis, California 95616, United States; ‡ Stanford Synchrotron Radiation Lightsource, SLAC National Accelerator Laboratory, Menlo Park, California 94025, United States; § Department of Viticulture & Enology, University of California, Davis, California 95616, United States

## Abstract

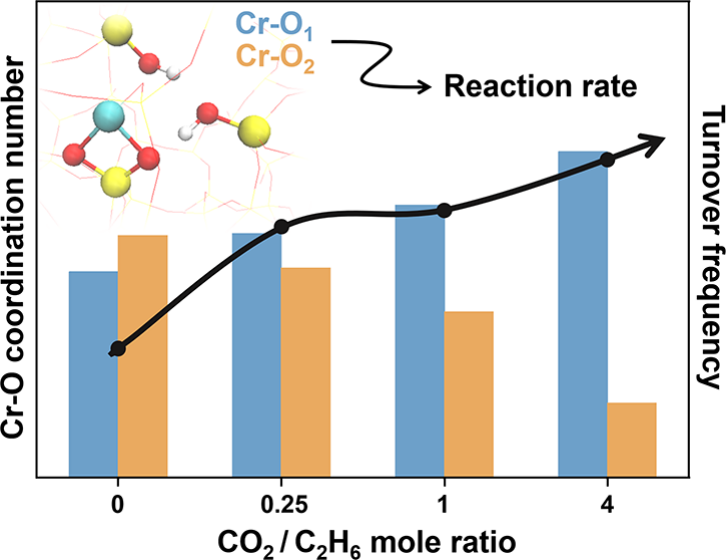

The reduction of CO_2_ is known to promote increased alkene yields from alkane dehydrogenations when the reactions are cocatalyzed. The mechanism of this promotion is not understood in the context of catalyst active-site environments because CO_2_ is amphoteric, and even general aspects of the chemistry, including the significance of competing side reactions, differ significantly across catalysts. Atomically dispersed chromium cations stabilized in highly siliceous MFI zeolite are shown here to enable the study of the role of parallel CO_2_ reduction during ethylene-selective ethane dehydrogenation. Based on infrared spectroscopy and X-ray absorption spectroscopy data interpreted through calculations using density functional theory (DFT), the synthesized catalyst contains atomically dispersed Cr cations stabilized by silanol nests in micropores. Reactor studies show that cofeeding CO_2_ increases stable ethylene-selective ethane dehydrogenation rates over a wide range of partial pressures. *Operando* X-ray absorption near-edge structure (XANES) and extended X-ray absorption fine-structure (EXAFS) spectra indicate that during reaction at 650 °C the Cr cations maintain a nominal 2+ charge and a total Cr–O coordination number of approximately 2. However, CO_2_ reduction induces a change, correlated with the CO_2_ partial pressure, in the *population* of two distinct Cr–O scattering paths. This indicates that the promotional effect of parallel CO_2_ reduction can be attributed to a subtle change in Cr–O bond lengths in the local coordination environment of the active site. These insights are made possible by simultaneously fitting multiple EXAFS spectra recorded in different reaction conditions; this novel procedure is expected to be generally applicable for interpreting *operando* catalysis EXAFS data.

## Introduction

Developing technologies to upgrade alternative feedstock molecules is an essential step toward reducing the reliance on petroleum for the production of commodity chemicals. In this context, ethane dehydrogenation is an attractive reaction system for ethylene production because ethane is a primary constituent of natural gas, and its direct conversion is potentially more carbon-efficient than traditional catalytic cracking systems.^1^ Current ethane dehydrogenation process economics are limited by low single-pass ethylene yields, which result from the prevalence of side reactions and catalyst deactivation by coke deposition.^2^ It has been observed that the use of CO_2_ as a coreactant enhances catalyst activity, facilitates high ethylene selectivity at high alkane conversion, and extends catalyst lifetime by reducing coking.^3−30^

The role and impact of CO_2_ on the reaction are highly dependent on the nature of the catalyst.^14^ When reducible oxide catalysts are used, ethane dry reforming can occur in parallel, and commonly the role of CO_2_ is to replace reacting lattice oxygen in a redox Mars–van Krevelen mechanism.^31^ When mononuclear metal centers are active sites, the role of CO_2_ may be to efficiently remove hydrides,^32^ which form through direct interaction of ethane with the metal site.^33^ In some instances, the role of CO_2_ addition is to simply increase stability through the reverse Boudouard reaction.^34^

In this work, we focus specifically on investigating the promotional effect induced by the reduction of CO_2_ to CO by hydrogen provided by ethane—that is, parallel or tandem CO_2_ reduction and ethane dehydrogenation (CO_2_–EDH) to produce ethylene, syngas, and water, where all CO originates from CO_2_ and the C–C bonds of the hydrocarbon remain intact. This system is particularly interesting because it produces a dry basis product composition of C_2_H_4_, H_2_, and CO, with the H_2_:CO ratio tunable based on the CO_2_:C_2_H_6_ feed ratio; this composition is attractive for downstream upgrading reactions such as hydroformylation.^35^ We sought to develop a catalyst platform that facilitates mechanistic studies under highly controlled, stable conditions, where the promotional effect of CO_2_ originates from the CO_2_ reduction reaction.

To isolate and understand the role of CO_2_ reduction in promoting alkane dehydrogenation, the enabling catalytic system should possess a number of characteristics: (i) contain a well-defined active-site environment; (ii) in the regime of differential ethane conversions, all ethane is dehydrogenated with no dry reforming side reactions; (iii) ethane conversion and product selectivity are stable over wide ranges of CO_2_ partial pressures; and (iv) CO_2_ is reduced to CO at rates commensurate with ethane dehydrogenation. Here, we show that atomically dispersed, cationic chromium stabilized in silanol nests within highly siliceous MFI zeolite is a material platform with these attributes. *Operando* and *ex situ* characterization of this catalyst are used to assess the role of CO_2_ reduction in promoting ethylene-selective ethane dehydrogenation. A primary finding of our study, enabled by *operando* X-ray absorption spectroscopy, is that the promotional effect of parallel CO_2_ reduction is tied to subtle changes in the local environment of the mononuclear Cr active site. Although during catalytic turnovers the isolated Cr cations maintain a nominal 2+ charge and an unchanging total Cr–O coordination number of approximately 2, CO_2_ reduction during ethane dehydrogenation induces a change in the *population* of two distinct Cr–O scattering paths (and therefore to bond lengths).

## Results and Discussion

### Synthesis and Characterization of Cr/Si-MFI

Highly dispersed Cr species in siliceous MFI were created through a multistep process. To maximize the homogeneity of Cr-stabilizing sites within the micropore environment of the support, a borosilicate MFI zeolite (referred to as B-MFI) post-synthesis substitution was applied (details in the Supporting Information). Homogeneous boron distributions are readily obtained through robust B-MFI synthesis procedures.^36,37^ Deboronation of the calcined B-MFI upon hydrochloric acid treatment (referred to as Si-MFI) has been reported to generate silanol nests with reproducible quantity and distribution;^38^ these silanol sites are highly thermally stabile and stabilize Cr cations.^39−42^ Chromium was incorporated onto Si-MFI through vapor-phase deposition of chromium(III) acetylacetonate (Cr(acac)_3_).^39,43,44^ Cr(acac)_3_ was chosen as the metal precursor because it was reported to be effective in depositing mononuclear Cr species onto oxide surfaces.^44^ Since the intact chromium acetylacetonate molecule is too large to diffuse through the zeolite pores, it is expected that the sublimation process grafts the precursor onto terminating silanols on the external surfaces of the MFI particles. During the subsequent high-temperature oxidative treatment, CrO_*x*_, formed from the decomposition of Cr(acac)_3_, is found here to be sufficiently mobile to migrate into the zeolite micropores (referred to as Cr/Si-MFI).

In order to simplify the dehydrogenation chemistry and maximize site uniformity, Cr/Si-MFI with a relatively low chromium loading (0.5 wt %) was prepared. The elemental composition of the material was determined by inductively coupled plasma mass spectrometry (ICP-MS) (Table S1). The Cr/Si-MFI sample contained 0.547 wt % chromium, which closely matches the targeted metal loading. X-ray diffraction patterns (Figure S1) of synthesized zeolites confirm the formation of the MFI structure, and the characteristic features were preserved after deboronation and the introduction of chromium. The lack of diffraction signal attributable to crystalline chromium oxides within the sensitivity of the measurement suggests a high dispersion of chromium species. N_2_ physisorption results are shown in Figure S2 and Table S2. The N_2_ adsorption isotherms for all samples display the same type of hysteresis loop, indicating the presence of mesoporosity. Deposition of Cr onto Si-MFI was not associated with any change in the Brunauer–Emmett–Teller (BET) surface area.

### CO_2_–EDH Catalytic Performance of Cr/Si-MFI

**Figure 1:**
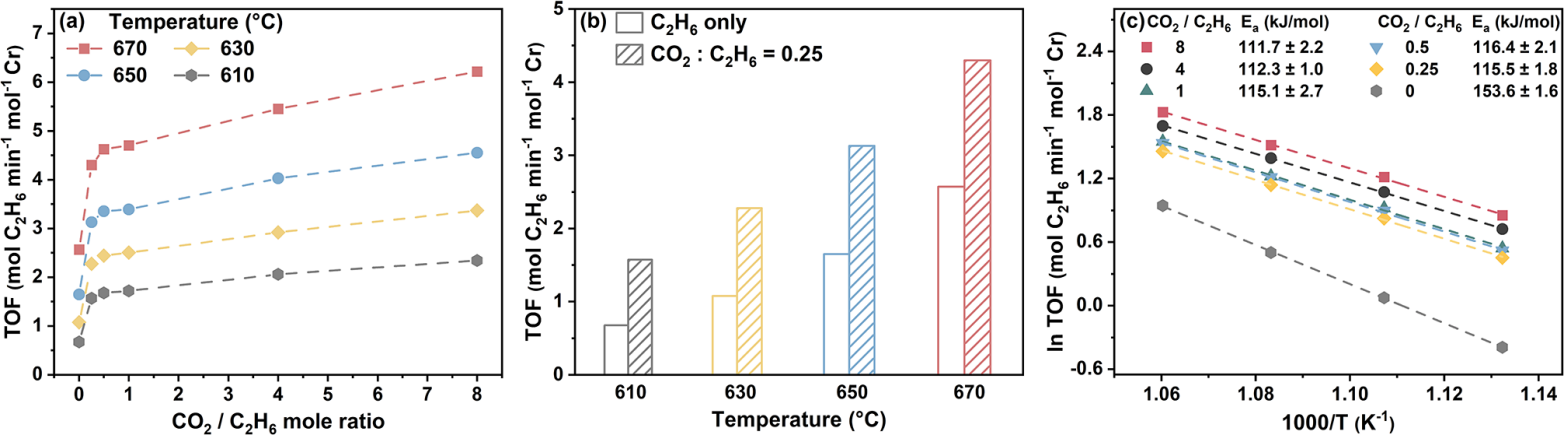
Results from CO_2_–ethane dehydrogenation with 0.5 wt % Cr/Si-MFI. (a) Rate of ethane conversion per Cr site vs CO_2_/C_2_H_6_ mole ratio at four reaction temperatures. (b) Comparison of ethane conversion rate with (filled bar) and without CO_2_ (empty bar) at four reaction temperatures. (c) Arrhenius plots corresponding to data in panel (a). Apparent activation energies labeled in the plot were calculated by fitting data to the Arrhenius equation. Reaction conditions: *T* = 610–670 °C; *P* = 2.5 psig; total flow rate: 48 sccm; C_2_H_6_ flow rate: 4 sccm; CO_2_ and N_2_ flow rates set based on the target CO_2_/C_2_H_6_ mole ratio; catalyst amount: 20–25 mg. The reported rates of ethane conversion per Cr site (turnover frequency; TOF) were calculated by averaging five data points collected during the initial 75 min of steady-state time on stream.

Kinetics studies were performed using a once-through packed-bed reactor to gain insight into the impact of CO_2_ on ethane dehydrogenation over Cr/Si-MFI catalysts. All measurements were operated in a differential regime with less than 10% ethane conversion to eliminate the influences of secondary reactions and product inhibition; confirmation of this condition is provided by the linear correlation between the ethane conversion and the amount of catalyst loaded (Figure S3). The influence of CO_2_ on ethane conversion was assessed by systematically varying the CO_2_ partial pressure and temperature during ethane dehydrogenation, with major results summarized in Figure 1. Ethane conversion was negligible in the presence of the bare Si-MFI support under all conditions examined (Table S3). To isolate the activity attributable to Cr sites, the rate measured for an equivalent mass of Si-MFI was subtracted from the rate measured for Cr/Si-MFI. Figure 1a provides the dependence of the ethane conversion rate on the CO_2_/ethane feed ratio (CO_2_ partial pressure) at temperatures from 610 to 670 °C. At low CO_2_ feed ratios (CO_2_/C_2_H_6_ < 0.5), ethane conversion rates increase significantly with an increasing mole fraction of CO_2_. Further increases in the cofed CO_2_ mole fraction (CO_2_/C_2_H_6_ > 1) are associated with diminishing improvements in dehydrogenation activity. Overall enhancements in the rate of ethane conversion were also observed as the reaction temperature increased. At all temperatures and feed conditions examined, including CO_2_ in the feed has a clear positive impact. Even with CO_2_/C_2_H_6_ as low as 0.25, the reaction rate was nearly twice that of the C_2_H_6_-only condition (Figure 1b). Apparent activation energies (*E*_a_) for ethane conversion under the same conditions were determined by fitting Arrhenius plots (Figure 1c) generated through the data collection methodology shown in Figure S4. For each investigated condition, the rate measured at 650 °C was used as a reference to determine whether the catalyst deactivated during data collection (Figure S4). The consistent rates measured at 650 °C before and after the temperature ramp (610–670 °C) confirm that Cr/Si-MFI was stable, and the data collected to determine *E*_a_ are reliable. The inclusion of CO_2_ in the feed yields lower apparent activation energies for ethane conversion (111.7 ± 2.2 to 116.4 ± 2.1 kJ/mol) than does the C_2_H_6_-only case (153.6 ± 1.6 kJ/mol). The apparent activation energies obtained for all conditions with CO_2_ are similar regardless of the feed ratio. These observations on the ethane conversion rate suggest that the dehydrogenation activity of the Cr/Si-MFI catalyst is promoted by the presence of CO_2_ and is tuned by changing the partial pressure. Though the similarity in *E*_a_ for CO_2_-included conditions implies that a further increase in CO_2_ may not further change the reaction pathway or the rate-determining step, an alteration in the structure or abundance of Cr active sites due to this CO_2_ feed ratio change could be responsible for the improved dehydrogenation activity.

For the reactor studies above, the product distribution was also examined to assess the influence of CO_2_ on the selectivity. Figure S5 provides the product selectivity as a function of temperature for multiple CO_2_/C_2_H_6_ feed ratios. Ethylene is the primary product, with a selectivity above 98% among all studied conditions. The steady-state product mole ratio of (H_2_ + CO)/C_2_H_4_ is close to unity throughout the experiments (Figure S6); this value is consistent with a system whose reaction stoichiometry is equivalent to that of ethane dehydrogenation to ethylene and reverse water gas shift occurring either in series or in a concerted mechanism, and negligible ethane dry reforming occurs. Small amounts of methane (0.12–1.78% selectivity) and propylene (0–0.14% selectivity) were detected as minor byproducts. They could be formed together through the cracking of products generated by ethylene oligomerization, and methane alone could be generated either from CO_2_ hydrogenation or from ethane steam cracking.^45^ Notably, with the improvement in the ethane conversion activity, only a slight decrease in the ethylene selectivity was observed as the CO_2_ feed ratio or reaction temperature increased.

In the presence of CO_2_, the Cr/Si-MFI catalyst was stable in differential regimes with ethane conversions below 10%: time on stream data showed no deactivation over 138 h while maintaining ethylene selectivity greater than 98% at 650 °C (Figure S7). Higher reaction temperatures (690 and 710 °C) were studied (Figure S4), and the CO_2_–EDH rates at these two temperatures were stable over 75 min on stream. The Cr/Si-MFI catalyst only deactivated irreversibly in the C_2_H_6_-only condition when the temperature was above 670 °C. These results show that CO_2_ plays a critical role in maintaining the catalytic performance. For dehydrogenation reactions, catalyst deactivation usually occurs through coke formation, active-site transformation, and metal active-site sintering.^46^ For this catalytic system, it is likely that CO_2_ protects the catalyst from deactivation through either (i) coke consumption by the reverse Boudouard reaction or (ii) by creating an environment that stabilizes the Cr active-site environment or its redox properties.

Taken together, the results from reactor studies show that the 0.5 wt % Cr/Si-MFI is an ideal platform to enable further study of the mechanistic role of cofed CO_2_ during ethane dehydrogenation—Cr active sites are dispersed, ethylene-selective, stable, and reaction rates are sensitive to the partial pressure of CO_2_ in the feed.

### Probing the Nature of Cr Sites in As-Synthesized Catalysts

**Figure 2:**
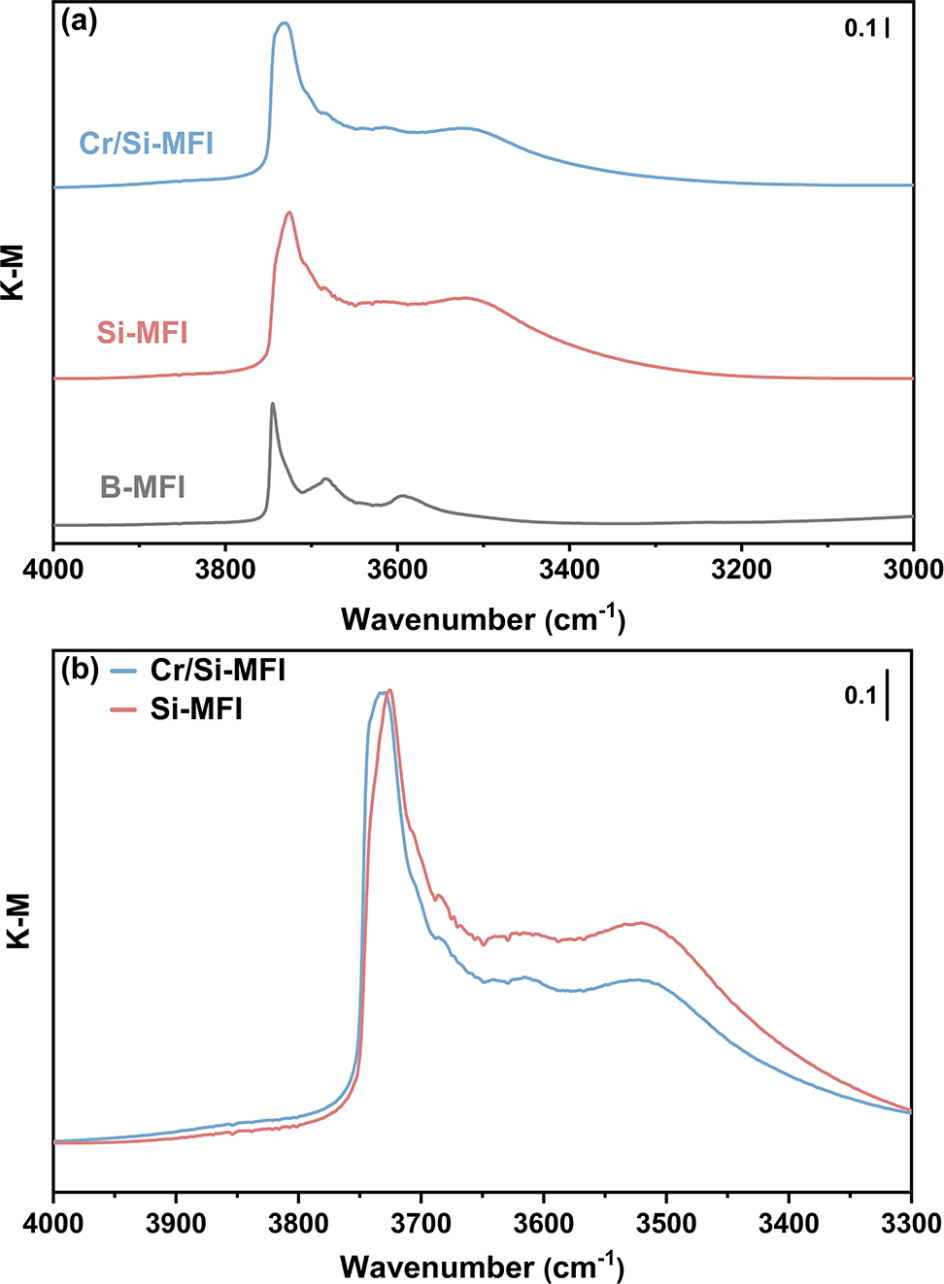
IR spectra in the O–H (ν_OH_) stretching region characterizing MFI samples. (a) Comparison among B-MFI (gray), Si-MFI (pink), and Cr/Si-MFI (blue) indicates the creation of silanol nests during deboronation. (b) Comparison between the same amounts of Si-MFI and Cr/Si-MFI shows the consumption of silanol nests after Cr introduction. All spectra were recorded at 100 °C in flowing N_2_ after holding at 350 °C for 1 h. In order to reflect the OH group band intensity change due to Cr introduction and eliminate the impact from different temperature treatments during synthesis, both Si-MFI and Cr/Si-MFI were *in situ*-calcined in flowing air at 600 °C for 6 h before the IR measurement.

The as-synthesized Cr/Si-MFI catalysts were characterized by using infrared spectroscopy. Figure 2 provides a comparison of IR spectra in the O–H stretching (ν_OH_) region characterizing MFI samples recorded at 100 °C under N_2_ flow. All samples were heated in flowing nitrogen at 350 °C for 1 h before the spectra were collected to eliminate the impact of moisture without altering zeolite hydroxyl groups. Similar to observations in previous reports, the spectrum characterizing B-MFI in Figure 2a exhibits three characteristic ν_OH_ bands: a sharp band at 3745 cm^–1^ (isolated silanol),^47−49^ a broad band at 3680 cm^–1^ (isolated B–OH groups or strong hydrogen-bonded B–OH groups neighboring Si–OH),^50−52^ and a broader band at 3590 cm^–1^ (weak hydrogen-bonded B–OH groups adjacent to hydroxyls).^52^ In the spectrum characterizing Si-MFI, a new intense and broad band centered at 3510 cm^–1^ is formed, which is a characteristic feature of hydrogen-bonded Si–OH groups.^53,54^ The B–OH band at 3590 cm^–1^ is not evident in the spectrum for the deboronated sample, but some intensity associated with the band at 3680 cm^–1^ is visible; this intensity is low enough that it is mostly obscured by the dominant Si–OH absorptions. These results are consistent with the removal of boron from the crystalline MFI framework and the creation of silanol nests (defect sites) upon deboronation with acid treatment. The IR spectrum of Cr/Si-MFI shows hydroxyl bands similar to that of Si-MFI. However, introducing Cr onto Si-MFI zeolite leads to an apparent intensity reduction in the bands associated with silanol nests, as shown in Figure 2b. In line with what has been observed for the incorporation of Cr onto dealuminated BEA zeolite,^55−57^ this result suggests that silanol nests have been consumed for metal reoccupation, and that most of the chromium has been preferentially anchored by these defects sites in the 0.5 wt % Cr/Si-MFI sample.

**Figure 3:**
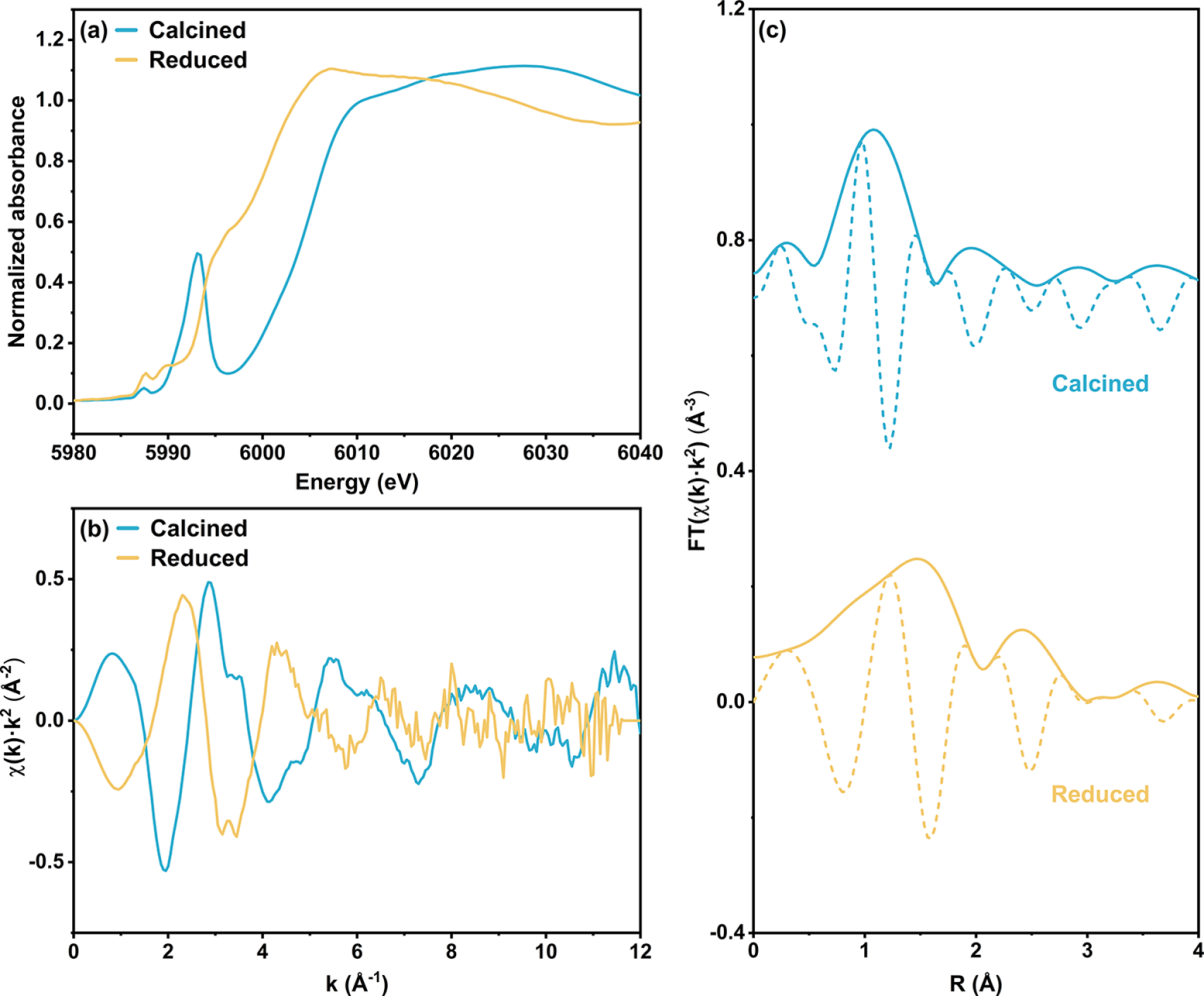
Redox behavior of Cr in Cr/Si-MFI: characterization by Cr K-edge XAS. Spectra of calcined Cr/Si-MFI were recorded in flowing helium at room temperature after calcination in flowing air at 600 °C for 6 h, while the reduced Cr/Si-MFI spectra were recorded during exposure to flowing H_2_ at 650 °C. (a) Normalized XANES spectra. (b) *k*^2^-weighted Cr K-edge EXAFS data. (c) Magnitude (solid) and imaginary components (dashed) of *k*^2^-weighted, phase-uncorrected Fourier transform of Cr K-edge EXAFS data.

Cr K-edge X-ray absorption spectra were collected to understand the oxidation state and local structure of the chromium site on the highly siliceous MFI zeolite support (Figure 3). Following high-temperature pretreatment in flowing air for 6 h, X-ray absorption near-edge structure (XANES) and extended X-ray absorption fine-structure (EXAFS) spectra of the as-synthesized sample were collected at room temperature in flowing helium (denoted as calcined). As shown in Figure 3a, the XANES spectrum of the calcined Cr/Si-MFI displays a prominent pre-edge peak at 5993.0 eV due to the dipole-forbidden transition from 1s to 3d orbitals, which is characteristic of noncentrosymmetric tetrahedral Cr(VI) species.^47,58−61^ A similar pre-edge peak is also observed in the reference spectrum of the Na_2_CrO_4_ compound (Figure S9), indicating that chromium supported by Si-MFI was predominantly present as tetrahedral Cr(VI) before ethane dehydrogenation catalysis. Consistent with previous studies,^62,63^ the corresponding *k*^2^-weighted Fourier-transformed EXAFS spectrum (Figure 3c) exhibits a dominant signal centered at 1.06 Å (phase-uncorrected), which is primarily associated with the shorter Cr=O double bonds. The second evident peak at 1.95 Å (phase-uncorrected) originates from the scattering from nearby O and Si atoms that are not directly chemically bound to Cr.^64^ The lack of a significant signal above 2.5 Å suggests the absence of a significant amount of polynuclear Cr species (although a small amount would be difficult to detect^65^) and predominantly the presence of isolated Cr(VI) in the calcined sample.

Since the dehydrogenation reaction provides a relatively reducing environment for the catalyst, spectra of H_2_-reduced samples (denoted as reduced) were recorded to approximate the reduction upper limit of the Cr site. Later in this report, these spectra will be compared and studied together with *operando* spectra recorded during the dehydrogenation reaction. Referring to Figure 3a, it is evident that exposure of the sample to flowing H_2_ at 650 °C resulted in a clear red shift in the absorption edge of the spectrum with respect to those of the calcined sample and the Cr_2_O_3_ reference (Figure S9), suggesting that the average oxidation state of chromium is lower than Cr(III) in the reduced Cr/Si-MFI. The sharp peak associated with the tetrahedral Cr(VI) species was replaced by a new pre-edge feature at 5996.0 eV (assigned to 1s to 4p orbital transition), which is considered a fingerprint for supported Cr(II).^21,62,66^ Moreover, the white line of the reduced-sample spectrum is almost featureless compared with that of the Cr_2_O_3_ reference spectrum (Figure S9), indicating the absence of a detectable amount of crystalline Cr_2_O_3_. Similar results have been shown for 0.5 wt % CO-reduced Cr/SiO_2_ Philips catalyst *in vacuo*, indicating that Cr(II) is the preferred species for low-loading Cr catalysts with highly siliceous supports after reduction treatments.^64,67^ Consistent with the larger ionic radius of Cr(II) with respect to that of Cr(VI), the scattering path distance of the first-shell Cr–O elongates after reduction, as reflected in the corresponding Fourier transform of the EXAFS spectrum (Figure 3c). Upon the disappearance of the scattering contribution of Cr=O double bonds, the new Cr–O signal shifts toward a greater distance of 1.47 Å (phase-uncorrected) with subtle asymmetry, which originates from two longer Cr–O single bonds^62^ (*vide infra*). The second peak centered at 2.41 Å (phase-uncorrected) is due to the contribution from Cr–(O)–Si.^68^

To derive structural characteristics of the Cr sites, EXAFS modeling was conducted to quantify coordination numbers and scattering path distances. Both conventional analysis with Artemis software in the Demeter package and a computational approach were applied in our study.

**Figure 4:**
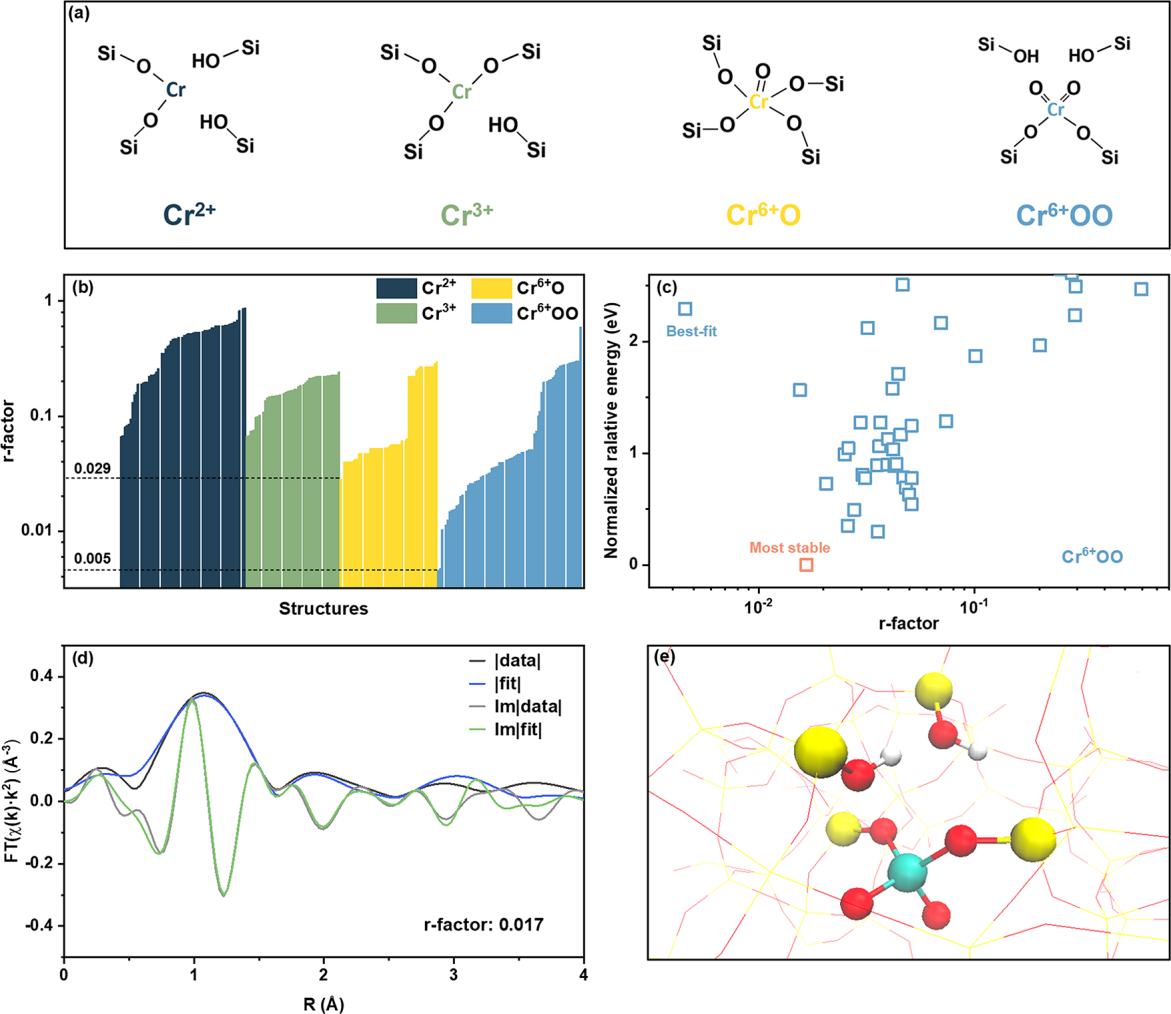
Local environment of chromium in calcined Cr/Si-MFI: theory-guided interpretation of EXAFS spectra. (a) Visual representation of chromium site motifs anchored by a single defect site. (b) r-factor distribution of 228 DFT-optimized structures generated based on Cr^2+^, Cr^3+^, Cr^6+^O, and Cr^6+^OO site motifs. (c) Normalized relative energy vs r-factor for all DFT-optimized structures generated based on the Cr^6+^OO site motif. The most stable structural model with a relatively good fit is labeled in salmon. (d) Magnitudes (fit, blue; experiment, black) and imaginary parts (fit, green; experiment, gray) of Fourier-transformed EXAFS of calcined Cr/Si-MFI with fitting based on the labeled most stable Cr^6+^OO structure in panel (c). (e) Visualization of the labeled most stable Cr^6+^OO structure corresponding to the fit shown in panel (d). Red, O; yellow, Si; white, H; green, Cr.

To elucidate the local environment of chromium in the calcined Cr/Si-MFI prior to catalysis, EXAFS spectra of calcined Cr/Si-MFI were fit with a theory-guided approach using the QuantEXAFS toolkit, which uses a database of density functional theory (DFT)-derived atomistic structures to fit experimentally measured EXAFS data. Compared to conventional EXAFS fitting approaches, QuantEXAFS is advantageous as the EXAFS fits are constrained by physically realistic structural models of the zeolite-confined active sites.^69^ The fitting procedure is described in detail in prior studies,^70,71^ and details related to this system are provided in the Supporting Information. Here, to select a set of structures for evaluation, chromium in 0.5 wt % Cr/Si-MFI is first assumed to be atomically dispersed and solely coordinated by the silanol nests, which is consistent with prior results in this weight loading regime.^68,72^ Four categories of atomically dispersed chromium sites anchored by a single silanol nest were evaluated, as shown in Figure 4a. These motifs are labeled according to the valence state and site geometry: Cr^2+^ for divalent chromium, Cr^3+^ for trivalent chromium, Cr^6+^O for hexavalent chromium with one Cr=O double bond, and Cr^6+^OO for tetrahedral hexavalent chromium with two Cr=O double bonds. Taking all 12 unique T-sites in the MFI framework and the possible variations of the same chromium site motif on a single T-site into account, a comprehensive library of chromium structures was generated using the MAZE package.^73^ Note that this approach inherently assumes that all of the chromium atoms occupy identical sites in the framework; accounting for the contributions from possible site heterogeneities is beyond the scope of this analysis.

In total, 228 DFT-optimized structures, including 62 Cr^2+^, 47 Cr^3+^, 48 Cr^6+^O, and 71 Cr^6+^OO, were considered. These structures were subsequently used to fit the experimental EXAFS spectrum, with the r-factor chosen to describe the goodness of the fit (the lower the r-factor, the better the fit). In agreement with the XANES interpretation of the calcined sample spectra above, the bar chart of r-factor distributions (Figure 4b) reveals that Cr^6+^OO sites, in general, are more representative of the calcined Cr/Si-MFI EXAFS spectra, as there are significantly more Cr^6+^OO structures with lower r-factor values — only fits using Cr^6+^OO structures yielded an r-factor below 0.029. In addition to the quality of the EXAFS fit, we also considered the relative stability of the Cr^6+^OO sites. Specifically, Figure 4c shows the normalized relative energies of all Cr^6+^OO structures (which are calculated from DFT using the RPBE-D3(BJ) functional^74,75^) plotted against the r-factor, where the relative energy is defined as the energy difference between the Cr^6+^OO structure on a certain T-site and the open-defect site structure on the same T-site. To allow a more straightforward comparison, the energy differences are referenced to the lowest energy structure. Since this energy term can be used to quantify the stability of the structure or the likelihood of the structure being formed, we choose to look at the more stable configurations with relative energies ≤ 2.6 eV compared to the most stable site.

Considering both the energetics determined by DFT and the quality of the EXAFS fits, we conclude that the Cr^6+^OO structure labeled in salmon in Figure 4c is the most representative and stable structural model of calcined Cr/Si-MFI, as it exhibits the lowest normalized relative energy and a reasonably good fit with an r-factor of 0.017. As shown in Figure 4e, the labeled most stable structure consists of tetrahedral hexavalent chromium anchored by a single-framework defect site and is located at the channel intersection. Through its comprehensive assessment of possible Cr sites, QuantEXAFS has resolved the location of the tetrahedral Cr(VI) that was evident from the XANES line shape. The corresponding theoretical Fourier-transformed EXAFS spectrum of this structure is shown in Figure 4d, which is in close agreement with the experimental data. The fitting results (Table S4) reveal that the first shell of the spectrum is assigned to Cr–O single scattering from two Cr=O double bonds (∼1.60 Å) and two Cr–O(−Si) single bonds (∼1.76 Å). The second shell mainly includes nearby Cr–O(−H) and Cr–Si path contributions. Though for the Cr^6+^OO site motif, there is a best-fit structure highlighted in Figure 4c with an r-factor of 0.005, this structure is unlikely to be formed or stabilized due to the high energy penalty. EXAFS fitting of the best-fit Cr^6+^OO structural model can be found in Figure S10. As recommended in our previous work,^69^ the entire database of DFT structures and their associated fits are provided in the Supporting Information.

Compared with the most stable Cr^6+^OO structural model fitting, the EXAFS spectra obtained using the other three types of chromium motifs do not match the experimental results well (Figure 4b). For instance, the best fits for Cr^2+^, Cr^3+^, and Cr^6+^O structural models (Figure S11) show significant deviations in the region of *R* greater than 1.7 Å that originate from the scattering contribution from longer-distance Si and O atoms.

### Dynamics of Cr Sites Captured by *Operando* XAS

The characteristics of the as-synthesized catalyst described above establish the initial state of the Cr sites but do not reflect changes associated with the CO_2_–EDH process. To relate the results from the above CO_2_–EDH catalysis studies to the properties of the isolated Cr sites, *operando* XAS experiments were performed on Cr/Si-MFI during CO_2_–EDH at 650 °C in flow conditions equivalent to those investigated in the packed-bed reactor measurements. To monitor the real-time structural evolution of the Cr site in response to the feed change, the reactant feed was adjusted from CO_2_-rich to CO_2_-deficient, with the CO_2_/C_2_H_6_ feed mole ratio decreasing from 4, 1, 0.25 to 0. As the CO_2_ partial pressure decreases, we expect a transformation in the reaction environment from moderately oxidative to reductive. Since H_2_ is a more potent reducing agent than ethane, the spectra recorded in flowing H_2_ at 650 °C (denoted as H_2_ only) are reported to provide a reference condition reflecting the most reduced state possible for the noted flow conditions and temperatures.

**Figure 5:**
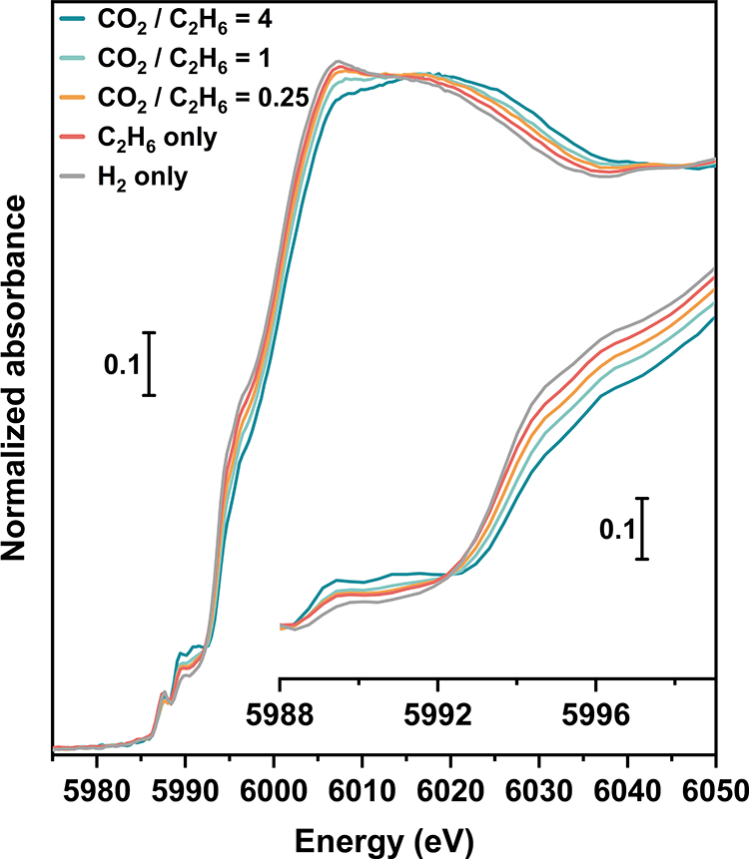
Normalized *operando* Cr K-edge XANES spectra of Cr/Si-MFI during the ethane (with/without CO_2_) dehydrogenation reaction at 650 °C. All spectra were collected through one continuous experiment, where the Cr/Si-MFI sample was exposed to CO_2_/C_2_H_6_/He mixture with a constant total flow rate of 66.4 sccm, and the C_2_H_6_ flow rate was maintained constant at 6.7 sccm. CO_2_ and He flow rates were adjusted based on the targeted CO_2_/C_2_H_6_ mole ratio, following the order of decreasing the CO_2_/C_2_H_6_ mole ratio from 4, 1, 0.25 to 0. For comparison, the spectrum of the H_2_ reduced sample is also reported, which was recorded in flowing H_2_ at 650 °C. The inset reports the magnification of the pre-edge features. The reoccurring glitch at 5987.5 eV was due to the Si (111) crystal sets used during measurements, and we have chosen not to deglitch the XAS spectra.

Figure 5 shows the feed-dependent modifications in the Cr K-edge XANES of Cr/Si-MFI during the ethane dehydrogenation reaction. Upon exposure of Cr/Si-MFI to ethane and CO_2_ at 650 °C, the reaction begins immediately with ethylene, water, and CO observed using the downstream mass spectrometer. All of the XANES spectra recorded under reactive conditions (denoted according to the flow conditions) show features similar to those of the H_2_-only spectrum. The prominent pre-edge component at 5993.0 eV assigned to the tetrahedral Cr(VI) species in the calcined spectrum is replaced by two pre-edge features at 5989.4 and 5990.6 eV (Cr_1s_ → Cr_3d_ + O_2p_). Another component, almost on the edge at 5996.0 eV (Cr_1s_ → Cr_4p_), is associated with supported Cr(II) species^47,66^ (inset of Figure 5). This result suggests that regardless of the CO_2_ feed, the Cr oxidation state remained nominally Cr(II) during the ethane dehydrogenation reaction.

Although nominally in the Cr(II) state, the overall blue shifts in the XANES spectra as the CO_2_ partial pressure increases do indicate an increasing average charge on the Cr sites. Although no stable relevant commercial Cr(II) reference compound is available, considering the evident red shifts in the edges of all *operando* spectra with respect to those of the Cr_2_O_3_ and Cr(acac)_3_ reference compounds (Figure S12), and the qualitatively similar features among the spectra (Figure S13), our analysis suggests it is unlikely that the blue shift results from a significant change in the abundance of the Cr^2+^/Cr^3+^ redox couple. A more likely origin of the observed blue shift is electron donation from Cr to a molecular adsorbate present under reaction conditions, such as carbon monoxide (CO) or a hydride (Cr–H). Since increasing CO_2_ partial pressure increases the turnover rate for CO_2_–EDH, the time-averaged concentration of adsorbates and intermediates also increases with CO_2_ partial pressure. This would tend to reduce the electron density, as reflected in the XANES features that suggest the slight oxidation of Cr sites. Based on the above discussion, we conclude that Cr(II) is the dominant active species in the Cr/Si-MFI catalyst during the CO_2_–EDH reaction.

This conclusion suggests that CO_2_ reduction must induce a subtle change in the electronic structure of the chromium active centers. Aside from the blue shift, the isosbestic points shown in Figure 5 are noteworthy, as they demonstrate a systematic and stoichiometric change in the ligand environment of Cr as the CO_2_ supply increases. To further investigate the dynamics of the local structure of the chromium site in response to CO_2_ partial pressure and CO_2_ reduction rate, Cr K-edge *operando* EXAFS spectra were recorded in the same reaction conditions simultaneously.

**Figure 6:**
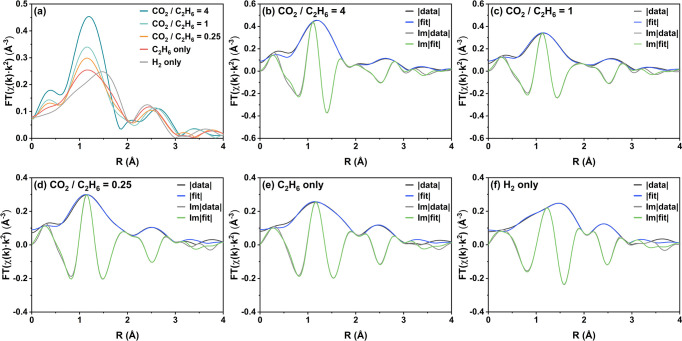
Local environment of chromium in Cr/Si-MFI during reactions: conventional interpretation of Cr K-edge *operando* EXAFS spectra through Artemis. (a) Magnitudes of the *k*^2^-weighted, phase-uncorrected Fourier transform of the EXAFS signal collected with the XANES spectra reported in Figure 5 (same symbols and reaction conditions used). (b–f) Magnitudes (fit, blue; experiment, black) and imaginary parts (fit, green; experiment, gray) of Fourier-transformed EXAFS of Cr/Si-MFI in flowing CO_2_/C_2_H_6_/He mixture or H_2_ at 650 °C. *k*-range of 2.9–8.0 Å^–1^ and *R*-range of 1.0–3.1 Å were chosen for the simultaneous fittings based on the best-fit model.

Consistent with the results from the *operando* XANES measurements, the magnitudes of the *k*^2^-weighted Fourier-transformed EXAFS spectra presented in Figure 6a exhibit similar trends, which are characterized by the well-defined first-shell peaks centered at around 1.16 Å (phase-uncorrected), the intensity of which decrease as the system is changed from CO_2_-rich to CO_2_-deficient. Although EXAFS alone is not capable of distinguishing between Cr–O and Cr–C contributions, the first-shell peaks here are assumed to result from chromium coordination with oxygen because the feature was present in the *ex situ* spectra in the absence of C-containing species (however, the presence of Cr–C species was considered as well; see below). In agreement with the result from XANES analysis that Cr sites were slightly oxidized during the CO_2_–EDH reaction, the first-shell peaks of the *operando* spectra are all shifted toward the lower *R* region when compared with that of the H_2_-only spectrum, suggesting the contribution of a Cr–O single bond that is shorter than the Cr–O bond in the H_2_-reduced Cr(II) compound. The second-shell peaks in the 2.0–3.1 Å range (phase-uncorrected) show magnitudes similar to that of the H_2_-only spectrum, indicating a feed-independent structural contribution; therefore, this feature is assigned to Cr–Si scattering. The lack of significant higher-shell contributions (Cr–Cr scattering) again indicates that chromium primarily exists as monomeric species in reaction conditions. Altogether, these results support the hypothesis that stoichiometric changes occurred in the chromium site’s local environment, especially in the O-ligation of the Cr active center, with increased CO_2_ partial pressure and CO_2_ reduction rate (Figure S8).

To quantify the changes to the Cr site coordination environment resulting from the CO_2_–EDH reaction, the *operando* EXAFS spectra were modeled using the Artemis software in the Demeter package.^76^ Since the *operando* spectra and the H_2_-only spectrum share features and were all recorded in reactive atmospheres at 650 °C, they were fit simultaneously to increase the degrees of freedom for the fitting parameter, as well as reduce statistical error (modeling details in the Supporting Information).

**Figure 7:**
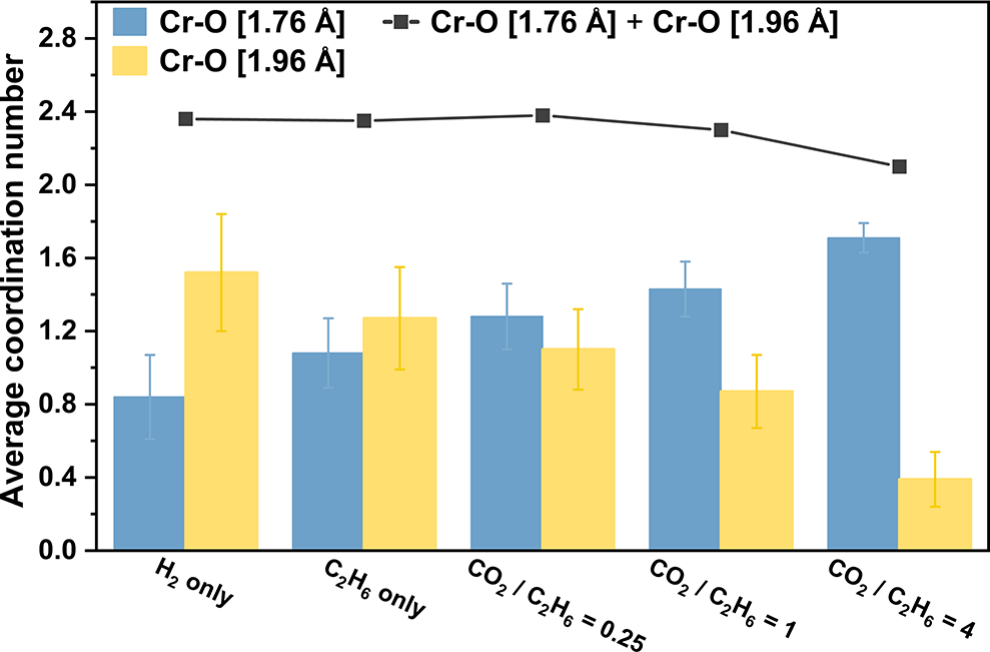
Comparison of Cr–O scattering paths in terms of the average coordination number, associated with EXAFS spectra recorded during reaction with different reactant feed ratios. Coordination number *N* fit results for Cr–O paths with scattering path lengths 1.76 Å (blue) and 1.96 Å (yellow) are based on the best-fit model, with error bars representing the statistical error of the fit.

Figure 6 and Table 1 summarize the EXAFS curve fitting results based on the best-fit model, with the following observations made:

(i) The EXAFS spectra characterizing the Cr/Si-MFI catalyst under various reactive conditions could only be precisely described by a best-fit model consisting of two Cr–O single scattering paths and one Cr–Si single scattering path, with effective scattering path lengths (*R*_eff_) of 1.79, 1.99, and 3.18 Å, respectively. As shown in Figure 6b–f and Figure S15, the calculated contributions based on the chosen model are in close agreement with all experimental spectra in the *k*-range of 2.9–8.0 Å^–1^ and *R*-range of 1.0–3.1 Å, giving an r-factor of 0.002. The fact that the best-fit model required two Cr–O scattering paths implies the coexistence of two different Cr–O bonds (or two types of O ligations). This claim is supported by the unsatisfactory fits that result from a model containing only one Cr–O scattering path, which are shown in Figures S16–S17 and Tables S6–S7. Our analysis also finds that any contribution from the Cr–C scattering path can be ignored, as proved by the unsatisfactory fits that result from a model containing an extra Cr–C path, shown in Figure S18 and Table S8.
(ii) Regardless of the reaction condition, the total average coordination number of Cr–O paths (*N*_Cr–O[1.79]_ + *N*_Cr–O[1.99]_) are in the range of 2.10–2.40 (Figure 7). Assuming chromium is covalently coordinated to oxygen only, this result is consistent with the conclusion based on XANES results that the average oxidation state of chromium sites is close to Cr(II) in the CO_2_–EDH reaction and H_2_-only conditions.
(iii) Regardless of the CO_2_ partial pressure, scattering path lengths *R* of the Cr–O and Cr–Si single scattering paths in general remain unchanged, with the first Cr–O bonding distance fit at 1.76 ± 0.01 Å, the second Cr–O bonding distance at 1.96 ± 0.01 Å, and the Cr–Si next-nearest-neighbor distance at 3.13 ± 0.01 Å (note that there are negligible decreases in the scattering path lengths in the C_2_H_6_-only and H_2_-only conditions).
(iv) The fit parameters of the Cr–Si path for five spectra are similar, indicating that the reaction condition does not significantly modify the coordination of framework silicon to the chromium sites.
(v) The measured disorder term (mean square disorder factor, σ^2^) of the Cr–Si path is larger than that of the Cr–O path, as is expected, reflecting a stronger interaction between chromium and oxygen.

**Table 1:** Summary of the Fit Parameters Representing Cr K-edge EXAFS Data Characterizing Cr/Si-MFI under Various Conditions^a^^,^^b^

Condition	Scattering path [*R*_eff_ (Å)]	*N*	*R* [Å]	Δ*E*_0_ [eV]^c^	10^3^·σ^2^ [Å^2^]^d^	*S*_0_^2^	r-factor
CO_2_/C_2_H_6_ = 4	Cr–O [1.79]	1.71 ± 0.08	1.76 ± 0.01	–4.17 ± 0.92	5.6	0.85^e^	0.002
	Cr–O [1.99]	0.39 ± 0.15	1.96 ± 0.01	–6.53 ± 2.94	5.5		
	Cr–Si [3.18]	1.12 ± 0.12	3.13 ± 0.01	–4.17 ± 0.92	10		
CO_2_/C_2_H_6_ = 1	Cr–O [1.79]	1.43 ± 0.15	1.76 ± 0.01	–4.17 ± 0.92	5.6		
	Cr–O [1.99]	0.87 ± 0.20	1.96 ± 0.01	–6.53 ± 2.94	5.5		
	Cr–Si [3.18]	0.97 ± 0.13	3.13 ± 0.01	–4.17 ± 0.92	10		
CO_2_/C_2_H_6_ = 0.25	Cr–O [1.79]	1.28 ± 0.18	1.76 ± 0.01	–4.17 ± 0.92	5.6		
	Cr–O [1.99]	1.10 ± 0.22	1.96 ± 0.01	–6.53 ± 2.94	5.5		
	Cr–Si [3.18]	0.84 ± 0.11	3.13 ± 0.01	–4.17 ± 0.92	10		
C_2_H_6_ only	Cr–O [1.79]	1.08 ± 0.19	1.75 ± 0.01	–4.17 ± 0.92	5.6		
	Cr–O [1.99]	1.27 ± 0.28	1.95 ± 0.02	–6.53 ± 2.94	5.5		
	Cr–Si [3.18]	0.96 ± 0.12	3.13 ± 0.01	–4.17 ± 0.92	10		
H_2_ only	Cr–O [1.79]	0.84 ± 0.23	1.73 ± 0.02	–4.17 ± 0.92	5.6		
	Cr–O [1.99]	1.52 ± 0.32	1.95 ± 0.02	–6.53 ± 2.94	5.5		
	Cr–Si [3.18]	0.85 ± 0.14	3.10 ± 0.02	–4.17 ± 0.92	10		

a *k*-range of 2.9–8.0 Å^–1^ and *R*-range of 1.0–3.1 Å were chosen for the simultaneous fittings based on the best-fit model.

b Notation: *N*, coordination number; *R*, scattering path length; Δ*E*_0_, energy correction factor; σ^2^, disorder term; *S*_0_^2^, passive electronic reduction factor.

c Δ*E*_0_ for Cr–O [1.79] and Cr–Si [3.18] scattering paths were constrained to be equal.

d σ^2^ for the Cr–O [1.79] scattering path of each spectrum was constrained to be equal, with the same applied to Cr–O [1.99] and Cr–Si [3.18] scattering paths. All σ^2^ terms were kept constant at the value of the first fit to decrease the statistical error.

e *S*_0_^2^ was set as 0.85 based on the modeling of the Cr foil EXAFS spectrum (Figure S14 and Table S5).

Although the sum of the coordination number of Cr–O paths under different conditions remains near 2, explicit dynamics within the O-ligation environment of the chromium site are indicated by the data. These dynamics are evident when comparing the coordination numbers associated with two different Cr–O scattering paths (Figure 7). As the reaction condition transitions from CO_2_-deficient (entirely reductive) to CO_2_-rich (moderately oxidative), the Cr–O path with a shorter scattering path length of 1.76 Å is favored, or more abundant, as reflected in the gradual increase of *N*_Cr–O[1.76]_ from 0.84 ± 0.23 in the H_2_-only condition to 1.71 ± 0.08 in the CO_2_/C_2_H_6_ = 4 condition. Conversely, a downward trend is shown during the same transition in the coordination number of the Cr–O path, with the longer scattering path length of 1.96 Å: from 1.52 ± 0.32 to 0.39 ± 0.15. These observed shifts in the Cr–O scattering paths are feed-dependent interconversions between two types of O-ligation, with the average coordination number interpreted as the proportion of the corresponding O-ligation. The gradual increase in the proportion of the shorter Cr–O [1.76 Å] coordination with increasing CO_2_ partial pressure is consistent with the overall blue shifts in the *operando* XANES spectra discussed above: the observed slight oxidation of monomeric chromium species is consistent with the valence electrons moving away from the metal center, which is associated with a decrease in ionic radius, thereby shortening the average Cr–O distance. Despite the noise in the *k*-space region beyond 8.0 Å^–1^, similar trends regarding Cr–O paths were also observed when the *k*-range of the fit was adjusted up to 9.0 and 10.0 Å^–1^, respectively, proving that the best-fit model is robust, and no other features were missed in the modeling (Figures S19–S20 and Tables S9–S10).

**Scheme 1:**
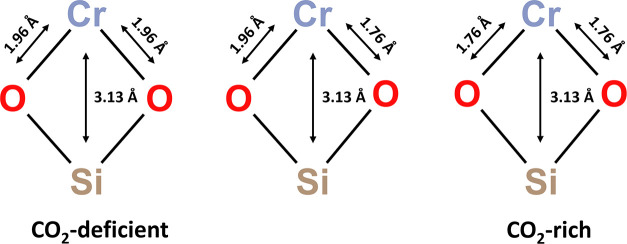
Depiction of the O-Ligation Population Shifting in Response to CO_2_ Partial Pressure for the Primary Active Cr(II) Sites during the Ethane Dehydrogenation Reaction.

Combining *operando* XAS with packed-bed reactor studies, these results demonstrate a correlation between ethane dehydrogenation activity and the microenvironment of isolated chromium active centers within the MFI zeolite framework. Scheme 1 provides a schematic illustration of Cr(II) as the primary active species during the ethane dehydrogenation reaction. Each Cr site is coordinated with two zeolite framework oxygen atoms. In the CO_2_-deficient regime, Cr sites are found to be relatively electron-rich and mainly coordinated to oxygen at a longer distance (1.96 Å). In the CO_2_-rich regime, which is associated with increased reaction rates, the charge on Cr increases slightly and the O-ligation population shifts toward greater amounts of the shorter Cr–O bond length (1.76 Å).

**Scheme 2:**
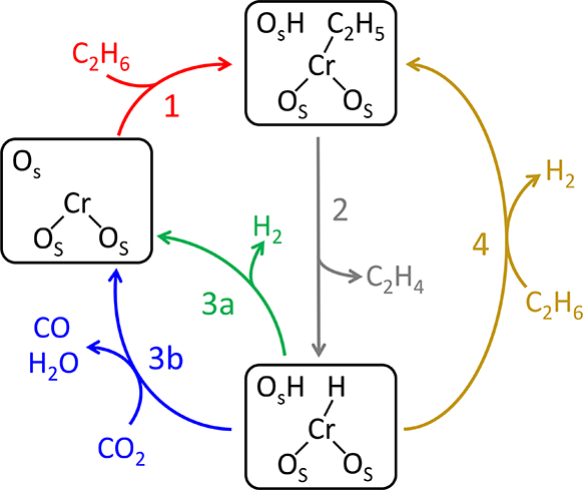
Proposed Catalytic Cycle for Ethane Dehydrogenation Motivated by Prior Studies of Silica-Supported Mononuclear Cr Catalysts,^33^ with the Potential Role of CO_2_ Reduction Added O_s_ represents a support surface oxygen. Only three O_s_ species are shown for brevity.

The results from reactor studies in the regime of kinetic control in Figure 1 reflect a decrease in the apparent activation energy for ethane conversion relative to direct dehydrogenation over the entire CO_2_ partial pressure range examined, which is consistent with previous CO_2_–EDH studies that the inclusion of CO_2_ into the ethane dehydrogenation pathway reduces the overall energy barrier.^8,30,77,78^ However, the similar *E*_a_, within error (about 115 kJ/mol), for all examined CO_2_ partial pressures implies that the CO_2_–EDH reaction pathway over Cr/Si-MFI is unchanging. Some conclusions exist in the literature regarding the reaction mechanism of ethane dehydrogenation over mononuclear Cr-based catalysts.^33,79,80^ As shown in Scheme 2, the reaction starts with the heterolytic C–H bond activation of ethane, forming Cr-ethyl (Cr–C_2_H_5_) and hydroxyl (−OH). A β-hydride transfer to chromium followed by ethylene desorption leaves a chromium hydride (Cr–H) and hydroxyl pair, and the whole catalytic cycle is completed through the recombinative evolution of H_2_ and regeneration of the original site. If this step does not occur readily, it is also possible for ethane activation to occur over the Cr–H bond. The rate-determining step for this process, which was reported to be either C–H bond activation or β-hydride transfer,^79,81^ is still debated.

Based on this reaction scheme, we considered the ways in which CO_2_ might alter the ethane dehydrogenation pathway and discuss here their likely relevance in the context of our results. One possibility is that CO_2_ directly causes oxidative modification of Cr sites, and the reaction proceeds through the Cr species redox cycle (e.g., Cr^2+^/Cr^3+^, Cr^3+^/Cr^6+^). The signature of this modification would be that the proportion of higher-valent Cr species would increase with the CO_2_ partial pressure, along with significant modification in the dehydrogenation kinetics. In such a scenario, as the conditions change from reducing to oxidizing, there should be a continuous blue shift in XANES spectra toward the absorption edge energy of Cr(III) or Cr(VI) and increases in the coordination of chromium to oxygen (*N*_Cr–O_). Results consistent with this hypothesis have been shown in previous reports, including an XAS investigation of 2.0 wt % Cr/SiO_2_ catalyst during oxidative propane dehydrogenation reaction (PDH), which reported the copresence of Cr(II) and Cr(III) species and their respective correlation to propane conversion and propene selectivity.^21^ Interestingly, the amount of isolated Cr^II^ cations was observed to decrease in favor of Cr^(III)^O_*x*_ oligomers, depending on the oxidizing character of the cofed oxidizers. In that study, a significant edge blue shift in *ex situ* XANES spectra was observed when the relative amount of Cr^(III)^O_*x*_ oligomers increased from 35 to 69%. By comparison, in our study, the blue shifts resulting from the CO_2_ partial pressure changes reflected in the *operando* XANES spectra are subtle (Figure S13), as are the changes among apparent activation energies for all CO_2_–EDH conditions. This indicates that it is the turnover of Cr active sites, but not the Cr valence, that changes upon dehydrogenation with variation in CO_2_ partial pressure and CO_2_ reduction rate (Figure S8). The observed lack of measurable Cr–Cr scattering and steady total Cr–O coordination indicates that oxidative modification, with significant changes in the abundance of a Cr redox couple, is unlikely for our system.

Therefore, the results indicate that coordinatively unsaturated Cr(II) is the primary active species in the Cr/Si-MFI catalyst during CO_2_–EDH. A plausible source for the enhanced dehydrogenation activity with the CO_2_ partial pressure is the accelerated turnover of Cr active sites for ethane or CO_2_ activation; in this scenario the reaction pathway remains unchanged. In agreement with previous theoretical and experimental studies on CO_2_-mediated dehydrogenations,^31,82^ CO_2_ directly reacts with an intermediate in Scheme 2, forming CO and facilitating H removal as H_2_O to complete the catalytic cycle. In contrast to the electrophilic addition activation of the C–H bond over Cr catalysts,^80^ the activation of CO_2_ usually involves electron transfer from the catalyst surface to the CO_2_ molecule, placing the formed CO_2_^–^ anion in a bent geometry, the dissociation of which is more energetically favored compared with a linear CO_2_ cation.^3^

As the reaction rate increases, the O-ligation population shifts toward favoring the shorter Cr–O bond length over the longer bond length, and the electron density around Cr centers decreases. The reaction rate is connected to the ligation population and charge through the time-averaged impact of adsorbates generated during the reaction cycle. This finding is consistent with a condition where the reaction of CO_2_ does not cause Cr to redox cycle among valence states but is capable of making Cr slightly more electron-deficient at steady state: the increased rate of adsorbate formation, by CO_2_ reduction, could shift electron density away from the Cr center, which manifests as a subtle blue shift in the XANES spectra. A recent study of Pt–Sn alloy catalysts during CO_2_-mediated propane dehydrogenation supports this scenario, where slight oxidation of platinum sites under reactive conditions was reported to originate from electron back-donation from the metal surface to the π* antibonding orbital of the adsorbed CO molecule.^83^

## Conclusions

This study has reported on the nature of highly dispersed Cr cations supported on a crystalline silicate zeolite (Si-MFI) and changes to the local structure of the Cr-associated active site during catalytic CO_2_-assisted ethane dehydrogenation. The outcomes of steady-state kinetic experiments were correlated with catalyst properties assessed through multiple spectroscopic characterization techniques, especially X-ray absorption spectroscopy. It was found that Cr, introduced by vapor-phase exchange, is highly dispersed and anchored by silanol nests within the micropores of Si-MFI. The catalyst is ethylene-selective and stable for CO_2_-assisted ethane dehydrogenation; parallel CO_2_ reduction was found to increase reaction rates over a range of partial pressures.

Cr K-edge X-ray absorption spectroscopy measurements in the EXAFS region recorded on the as-synthesized (calcined in O_2_) sample were analyzed using fits derived from density functional theory-optimized structures (QuantEXAFS). The results indicate that vapor-phase exchange followed by calcination yields tetrahedral Cr(VI) predominantly located at the MFI zeolite channel intersection. *Operando* X-ray absorption spectroscopy, performed during CO_2_-assisted ethane dehydrogenation at atmospheric pressure and 650 °C, generated results on the transformations of the Cr sites as a function of the CO_2_ partial pressure. Analysis of the EXAFS region indicated that the isolated Cr sites maintain a nominal 2+ charge and a steady total Cr–O coordination number of approximately 2 during catalytic turnovers. Reduction of CO_2_ to CO induces a change, correlated with the CO_2_ partial pressure, in the *population* of two distinct Cr–O scattering paths (Cr–O ligations). These results were derived through a novel interpretation of *operando* EXAFS data that is based on the simultaneous fitting of multiple spectra recorded in different reaction conditions; this procedure is expected to be generally applicable for the interpretation of *operando* EXAFS data for catalysis. Our findings demonstrate that the promotional effect of CO_2_ reduction on ethane dehydrogenation activity results from a subtle change to the local atomic environment around isolated Cr(II) active centers.

## Supporting Information Available

Demo script (ZIP)
Cr^6+^OO database (ZIP)
Experimental methods, computational methods, XRD of MFI samples, elemental composition of samples, N_2_ physisorption isotherms, N_2_ physisorption results, additional packed-bed reactor measurement data, XANES profiles, and EXAFS fitting results in Supporting Figures S1–S20 and Supporting Tables S1–S10 (PDF)
