# Supplementary material for: Reduction of Cofed Carbon Dioxide Modifies the Local Coordination Environment of Zeolite-Supported, Atomically Dispersed Chromium to Promote Ethane Dehydrogenation
Source: J Am Chem Soc. 2024 Mar 29;146(14):10060–72. doi: 10.1021/jacs.4c00995 (PMC11009955; doi:10.1021/jacs.4c00995)
Supplement: Supplementary file 3 — ja4c00995_si_003.pdf [file ja4c00995_si_003.pdf]

Supporting Information

Reduction of Cofed Carbon Dioxide Modifies the Local Coordination Environment of Zeolite-Supported, Atomically Dispersed Chromium to Promote Ethane Dehydrogenation

Wenqi Zhou[1], Noah Felvey[1], Jiawei Guo[1], Adam S. Hoffman[2], Simon R. Bare[2], Ambarish R. Kulkarni[1], Ron C. Runnebaum[1,3]*, Coleman X. Kronawitter[1]*

[1]Department of Chemical Engineering, University of California, Davis, CA 95616, United States

[2]Stanford Synchrotron Radiation Lightsource, SLAC National Accelerator Laboratory, Menlo Park, CA 94025, United States

[3]Department of Viticulture & Enology, University of California, Davis, CA 95616, United States

*Corresponding Authors

Table of Contents

1. **Experimental Methods**
2. **Computational Methods**
3. **Supporting Tables and Figures**

Figure S1. XRD of MFI samples

Table S1. Elemental composition of MFI samples

Figure S2. N_2 physisorption isotherms

Table S2. N_2 physisorption results

Figure S3. Ethane conversion and ethylene yield measured in the kinetic-controlled regime

Table S3. Ethane conversion summary of blank reactor tests

Figure S4. Time-on-stream rate of ethane conversion under different feed conditions

Figure S5. Product distribution of ethane dehydrogenation over Cr/Si-MFI

Figure S6. Time-on-stream product distribution under different CO_2 / C_2H_6 feed conditions

Figure S7. Time-on-stream ethane conversion and ethylene selectivity over 120 h

Figure S8. Rate of CO_2 conversion per Cr site vs CO_2 / C_2H_6 mole ratio

Figure S9. XANES characterizing Cr reference compounds

Table S4. Theoretical EXAFS fit parameters – the most stable $Cr^{6+}OO$ structural model

Figure S10. Theoretical EXAFS fitting of the best-fit $Cr^{6+}OO$ structural model

Figure S11. Theoretical EXAFS fitting of the best-fit Cr^{2+}, Cr^{3+}, and $Cr^{6+}O$ structural models

Figure S12. Comparison with Cr(III) reference compounds in XANES

Figure S13. Edge feature comparison between Cr reference compounds and Cr/SiMFI sample in reactive conditions

Figure S14. EXAFS fitting of spectrum characterizing Cr foil

Table S5. Best-fit EXAFS model – Cr foil

Figure S15. *k*-space fitting of spectra characterizing Cr/Si-MFI with *k*-range of 2.9–8.0 $Å^{-1}$

Figure S16. EXAFS fitting of rejected model containing Cr-O [1.99 Å] and Cr-Si [3.18 Å] paths

Table S6. Rejected EXAFS model containing Cr-O [1.99 Å] and Cr-Si [3.18 Å] paths

Figure S17. EXAFS fitting of rejected model containing Cr-O [1.79 Å] and Cr-Si [3.18 Å] paths

Table S7. Rejected EXAFS model containing Cr-O [1.79 Å] and Cr-Si [3.18 Å] paths

Figure S18. EXAFS fitting of rejected model containing Cr-O [1.79 Å], Cr-O [1.99 Å], Cr-Si [3.18 Å] and extra Cr-C [2.07 Å] paths

Table S8. Rejected EXAFS model containing Cr-O [1.79 Å], Cr-O [1.99 Å], Cr-Si [3.18 Å] and extra Cr-C [2.07 Å] paths

Figure S19. EXAFS fitting of spectra characterizing Cr/Si-MFI with *k*-range of 2.9–9.0 $Å^{-1}$

Table S9. Best-fit EXAFS model – *k*-range of 2.9–9.0 $Å^{-1}$

Figure S20. EXAFS fitting of spectra characterizing Cr/Si-MFI with *k*-range of 2.9–10.0 $Å^{-1}$

Table S10. Best-fit EXAFS model – *k*-range of 2.9–10.0 $Å^{-1}$

4. References

1. Experimental methods

1.1 Sample preparation

Synthesis of B-MFI. B-MFI zeolite was prepared based on a reported method.[1] 1.73 g sodium hydroxide (NaOH) and 3.55 g tetrapropylammonium bromide (TPABr) were added to 18.39 g deionized water separately and stirred until well dissolved. The NaOH solution was added to the TPABr solution and stirred for one hour. 25 g colloidal silica solution (40% SiO_2 in water) was added to the resulting mixture dropwise under vigorous stirring. Next, a solution of 0.82 g boric acid (H_3BO_3) in 8.2 g H_2O was added to the NaOH-TPABr-SiO_2 mixture, covered, and stirred overnight. The resulting aged gel with molar composition 1 Si : 0.08 B : 0.26 Na : 0.08 TPA : 20 H_2O was transferred to a 120 mL PEFT lined autoclave, sealed, and placed into an oven at 170 °C, rotating at 30 rpm for two days. After cooling the autoclave, the solid product was separated through centrifugation and washed with deionized water several times. The rinsed zeolite was dried in an oven at 120 °C overnight in ambient air and calcined in flowing O_2 at 600 °C to remove organics. Next, the sample was ammonium exchanged in aqueous 1 M NH_4NO_3 and calcined again in O_2 at 550 °C to convert it to its protonic form before use.

Preparation of Si-MFI. Si-MFI was prepared by treating the as-prepared B-MFI with acid. 1 g B-MFI and 50 mL 1 M HCl solution were added into a sealed, pressure-rated glass vessel, and the mixture was stirred and heated in an oil bath at 90 °C for 20 h. The solid product was separated by centrifugation and washed thoroughly with deionized water. The rinsed product was dried in an oven at 80 °C overnight in ambient air and was further dehydrated and calcined at 350 °C in flowing O_2. The as-prepared Si-MFI was transferred into the glovebox before use.

Deposition of Cr(acac)$_3$. Chromium was dispersed onto Si-MFI zeolite through vapor deposition of chromium(III) acetylacetonate (Cr(acac)$_3$). All steps were performed without sample exposure to ambient air and are described as follows: Si-MFI zeolite (up to 500 mg) and an appropriate amount of Cr(acac)$_3$ were added into a 2 mL glass ampoule. The ampoule was transferred and connected to a Schlenk line, where it was evacuated three times and flame-sealed under pressure below 100 mtorr. The sealed ampoule containing the light purple mixture was heated in furnace at 220 °C for 4 h, and 330 °C for 4 h, with a temperature ramping rate 5 °C/min. The sample color changed to green during this process. Next, the sample was transferred back to the glovebox, where it was removed from the ampoule and placed into a quartz calcination tube.

The sample in the calcination tube was air-free transferred to the furnace, heated under 40 mL/min air flow at 5 °C/min to 120 °C for 1 h, and 2 °C/min to 600 °C, at which temperature the furnace was held for 6 h. After cooling down, the Cr/Si-MFI sample was transferred back to the glovebox and stored before use.

1.2 Characterization methods

Physicochemical characterization. X-ray diffraction (XRD) was performed using a Bruker D8 Advanced Diffractometer with Cu Kα radiation. Sample elemental compositions were determined by inductively coupled plasma optical emission spectroscopy (ICP-OES) at Galbraith Laboratories, Inc. Nitrogen physisorption measurements were performed using a Micromeritics 3Flex Physisorption/Chemisorption Instrument. All samples were degassed under vacuum at 350 °C for 8 hours before N_2 adsorption, and nitrogen adsorption isotherms were recorded at -196 °C. Surface areas were determined by the BET method, and micropore volumes were determined by the t-plot method.

IR spectroscopy. Diffuse reflectance infrared Fourier transform spectra (DRIFTS) were collected using a Bruker Tensor II instrument equipped with Pike Technologies DiffusIR accessory. Spectra were collected using 256 scans with 2 cm^{-1} resolution. A spectrum of annealed CaF_2 under N_2 flow at room temperature was used as background, and the absorbance data was recorded using the Kulbelka-Munk equation. Zeolite samples were prepared for DRIFTS by dropping sieved pure sample powder (up to 5 mg) onto the top of a pile of CaF_2 in a ceramic sample cup. The loaded sample cup was placed into a Pike Technologies DiffusIR Heated Chamber inside the glovebox to allow air-free transfer to the spectrometer.

Sample treatments were performed in the DRIFTS heated chamber. The chamber was purged with 30 mL/min N_2 (Airgas, Research Purity) for 5 min at room temperature. Then the temperature was increased at 5 °C/min to 120 °C, where it was held for 1 h to remove adsorbed moisture. Next, samples were further heated at 5 °C/min to 350 °C and held at this temperature for 1 h. Spectra of the hydroxyl stretching region were taken when the chamber cooled back to 100 °C.

X-ray Absorption Spectroscopy. X-ray absorption spectroscopy (XAS) measurements were performed on the beam line 4-3 at the Stanford Synchrotron Radiation Lightsource (SSRL). Photon energy selection was achieved by a liquid nitrogen-cooled Si (111) monochromator. A

cylindrically bent Rh-coated mirror up-beam of the monochromator was used to collimate the X-ray beam and reject harmonics. The mirror was pitched to create a 10 keV cutoff. X-ray absorption near-edge structure (XANES) and extended X-ray absorption fine-structure (EXAFS) spectra were recorded at the Cr K-edge (5989.0 eV) in fluorescence mode using a PIPS detector. The incident beam was monitored using a nitrogen filled ionization chamber with a 1400 VDC bias and negative polarity. The chromium foil reference was scanned simultaneously with the sample for energy calibration. The Cr foil was mounted off-axis using Kapton tape to scatter some of the incident beam into the foil. A photo-diode was used to measure the transmitted beam through the Cr foil.

***In-situ* and *operando* XAS experiment.** The conditions of the *in-situ* and *operando* XAS experiments for Cr/Si-MFI were described as follows. Inside the glove box, the calcined Cr/Si-MFI catalysts (approximately 40 mg, as described in the above sample preparation section) were pressed into one pellet and loaded into an engineered *in-situ* XAS quartz tube cell, with a thermal couple nearby the sample pellet to monitor the temperature. The cell was sealed and air-free transferred to the beamline and connected to gas lines pre-purged with helium, avoiding sample exposure to ambient air. With the sample in flowing helium (50 mL/min) at room temperature, EXAFS spectra were collected (2 scans) and denoted as calcined. Next, the sample pellet was heated under helium (50 mL/min) at the rate of 5 °C/min to 650 °C, during which XANES spectra were collected continuously. Then, the temperature was kept at 650 °C for all *operando* XAS measurements: the sample pellet was exposed to CO_2 / C_2H_6 / He mixture with a total flow rate of 66.4 mL/min. The C_2H_6 flow rate was maintained at 6.7 mL/min to achieve constant ethane partial pressure. Flow rates of CO_2 and helium were adjusted based on the targeted CO_2 / C_2H_6 mole ratio, following the order of decreasing CO_2 / C_2H_6 mole ratio from 4, 1, 0.25 to 0. EXAFS spectra (9 scans) were collected and averaged to improve the signal-to-noise ratio for each condition. These spectra were denoted according to the flow condition. Finally, the gas flow was switched to H_2 / He mixture (6.7 mL/min H_2, 60 mL/min He), and EXAFS spectra (9 scans) were collected, denoted as H_2-only or reduced. Throughout the experiment, the effluent gas flow was continuously monitored by a downstream online mass spectrometer.

The Cr K-edge XANES spectra of reference compounds in Figure S9 were replotted from our previous reported results[2], which were also collected on Beamline 4-3 at SSRL in fluorescence mode. Reference samples were loaded into a stainless-steel flow cell equipped with cartridge heaters and Kapton windows, and spectra were recorded in ambient air at room temperature.

XAS data analysis. Raw XAS data was energy-calibrated, merged, and normalized using the Athena software, part of the Demeter package[3]. All spectra were energy-calibrated through the alignment of the Cr foil reference spectrum (collected with the sample spectrum simultaneously) to a pre-calibrated Cr foil spectrum at 5989.0 eV, with two to nine spectra merged to improve the signal.

For EXAFS data analysis, the EXAFS data was extracted in k-space, with the Fourier transform conducted on the k^2-weighted EXAFS function. Phase shifts and amplitudes for relevant backscattering were calculated using FEFF6. The Artemis software of the Demeter package was used for EXAFS modeling[3], considering k^1-, k^2-, and k^3-weighting. Considering the *operando* spectra and the H_2-only spectrum were all taken in reactive atmospheres at 650 °C, these five spectra (including CO_2 / C_2H_6 = 4, CO_2 / C_2H_6 = 1, CO_2 / C_2H_6 = 0.25, C_2H_6-only, H_2-only) were modeled simultaneously to increase the degree of freedom for the fit parameters and reduce the statistical error of each parameter. The maximum number of parameters extracted from the data was determined using the Nyquist criterion, with k-range of 2.9–8.0 $Å^{-1}$ and R-range of 1.0–3.1 Å.

For the EXAFS modeling of five spectra taken at 650 °C, the best-fit model consists of two Cr-O single scattering paths and one Cr-Si single scattering path. The first Cr-O and Cr-Si paths were obtained from a $Cr(Si_2O_5)_2$ cif file (mp-779017, from the Materials Project[4]), with effective scattering path lengths (R_{eff}) of 1.79 and 3.18 Å, respectively. The second Cr-O scattering path was obtained from a CrO cif file (mp-755073, from the Materials Project[4]) with R_{eff} of 1.99 Å. Fitting parameters for simultaneous modeling were set as follows:

(1) The passive electronic reduction term S_0^2 was fixed at 0.85 based upon the modeling of the Cr foil EXAFS spectrum (shown in Figure S14 and Table S5), using the first two Cr-Cr single scattering paths from a Cr metal cif file (mp-90, from the Materials Project).

(2) All spectra share the same E_0 values. Independent of the spectrum, all Cr-O [R_{eff} 1.79 Å] and Cr-Si [R_{eff} 3.18 Å] paths were set to share the same energy correction term ΔE_0 (denoted as E_1) since these two paths come from the same Cr reference cif file $Cr(Si_2O_5)_2$; all Cr-O [R_{eff} 1.993 Å] paths were set to share another ΔE_0 (denoted as E_2), as this path was imported from the CrO cif file, based on the assumption that the change in the edge position of the sample spectra to the reference spectrum is constant.

(3) Independent of the spectrum, all Cr-O [R_{eff} 1.79 Å] paths were set to share the same disorder term σ^2 (denoted as ss_1); Cr-O [R_{eff} 1.99 Å] paths were set to share the same σ^2 (denoted as ss_2);

Cr-Si [R_{eff} 3.18 Å] paths were set to share the same σ^2 (denoted as ss_3), based upon the assumption that all single-scattering paths at a similar distance would have a similar disorder term.

(4) Each path fit had unique coordination number N and ΔR terms for a given spectrum.

(5) The disorder terms σ^2 shown in Table 1 were kept constant at the value of the first fit to decrease the independent fit variants and statistical errors.

Limited by the maximum number of the independent parameters extracted from a single spectrum, the EXAFS spectrum of the calcined sample recorded at room temperature was modeled through a DFT-based approach (QuantEXAFS fit). The method details are described below in the Computational Methods section.

1.3 Catalytic performance measurements

Catalytic performance measurements were conducted in a quartz, packed-bed, down-flow reactor (4 mm inner diameters). Cr/Si-MFI sample powders were sieved between mesh sizes 60-40 before use and loaded into the quartz tube reactor between two lengths of α-alumina powder (2 g each) inside the glove box. Quartz wool was placed below the catalyst bed comprising zeolite sample and alumina to situate the catalyst bed at the furnace heated zone center. Both ends of the quartz tube reactor were connected to ultra-torr compression fittings, which allow air-free transfer. The reactor effluent was measured online by an Agilent 7890a gas chromatography (GC) unit equipped with FID and TCD detectors.

Prior to the CO_2-ethane dehydrogenation activity measurement, typically around 20-25 mg Cr/Si-MFI zeolite sample was heated at 5 °C/min to 650 °C in flowing N_2 (Praxair, 99.999%) at a flow rate of 40 mL/min. When the furnace temperature reached 650 °C, gas flow was switched to bypass the reactor in order to initialize and stabilize gas flow rates and composition for the dehydrogenation reaction. The reactor spent about 18 min under static N_2 at 650 °C before the reaction. During the dehydrogenation activity test at 650 °C, a mixture of C_2H_6 (Matheson, research purity), CO_2, and N_2 was fed through the quartz reactor at a total flow rate of 48 mL/min. Unless otherwise specified, the C_2H_6 flow rate was maintained at 4 mL/min for constant ethane partial pressure; CO_2 and N_2 flow rates were adjusted based on the targeted CO_2 / C_2H_6 mole ratio. Catalyst bed pressure was 2.5 psig. By varying the mass of the catalyst in the reactor (5-25 mg) and keeping flow conditions constant, the impact of space velocity at 650 °C was investigated. For

the experiments determining the reaction kinetics, the reaction temperature range was set from 610 to 670 °C, with the flow conditions kept the same.

Blank reactor experiments of 4 g α-alumina (with no catalyst) were run, the result of which were subtracted from the measured ethane conversions before calculating the rate of reaction. Unless otherwise specified, ethane conversion was maintained below 10% to achieve a differential reactor yielding accurate reaction rates. Reaction parameters were calculated as follows:

$$Ethane\ conversion = \frac{molar\ flow\ rate\ of\ carbon\ (C)\ in\ products}{molar\ flow\ rate\ of\ total\ C\ in\ efluent\ (\mathrm{C_2H_6}\ + products)} \times 100\%$$

$$Product\ i\ selectivity = \frac{molar\ flow\ rate\ of\ C\ in\ product\ i}{molar\ flow\ rate\ of\ C\ in\ all\ products} \times 100\%$$

$$Space\ velocity = \frac{molar\ flow\ rate\ of\ ethane\ in\ feed}{moles\ Cr\ in\ catalyst\ bed}$$

$$Rate\ of\ ethane\ converison = ethane\ converison\ \times space\ velocity$$

Note: Product selectivity is calculated on a dry and CO-free basis. The products in the above equation are referred to C_1-C_4 hydrocarbon species.

2. Computational methods

2.1 Initial structure generation

A comprehensive library of all geometrically unique structures of isolated chromium anchored by single defect sites varying in valence states was generated using the Multiscale Atomic Zeolite Simulation Environment (MAZE) package. This ensures the systematic and subjective generation of the initial structures close to their fully optimized geometries and significantly reduces the computational cost of constructing an exhaustive DFT database. Details for the initial structure generation are as follows:

(1) Divalent chromium, denoted as Cr^{2+}: starting from an open defect site with four Si-O(H), two out of four Si-O(H) were picked to bind Cr. Overall, such a site has 6 (4 combination 2) variations for each unique T-sites in the MFI topology.
(2) Trivalent chromium, denoted as Cr^{3+}: starting from an open defect site with four Si-O(H), one out of the four Si-O(H) was capped, whereas the remaining three Si-O(H) were used to bind Cr. Overall, such a site has 4 (${}_4C_1$ or ${}_4C_3$) variations for each unique T-sites.
(3) Hexavalent chromium with one Cr=O bond, denoted as $Cr^{6+}O$: starting from an open defect site with four Si-O(H), all four Si-O(H) were used to bind Cr. Instead of random generation of the initial position near Cr, the additional oxygen forming the Cr=O bond was inserted along the O(Si)-Cr vectors to introduce more diversity in the structure database used for the EXAFS fitting. Overall, there are 4 (${}_4C_1$ or ${}_4C_3$) variations for each unique T-sites.
(4) Hexavalent chromium with two Cr=O bonds, denoted as $Cr^{6+}OO$: starting from an open defect site with four Si-O(H), two Si-O(H) were used to bind Cr, and the remaining two were capped. Then, two additional oxygen were inserted near Cr, forming Cr=O bonds. The insertion direction was generated automatically through a Monte Carlo-based approach without human bias by finding a position near the Cr sites within 1.7 Å while not overlapping with any other framework atoms. Overall, there are 4 (${}_4C_1$ or ${}_4C_3$) variations for each unique T-sites.

2.2 DFT Calculations

All periodic DFT calculations were performed using the Vienna Ab-initio Simulation Package (VASP)[5–7] with a plane-wave cut-off of 400 eV and the revised-Perdew-Burke-Ernzerhof (RPBE) functional[8,9]. Dispersion interactions were included using the Grimme D3 method with Becke-Jonson damping[10,11], and a 0.03 eV/A force cut-off on each atom was used for geometry

optimization. The initial pure-silica ZSM-5 topology was obtained from the International Zeolite Association (IZA) database and was unit-cell optimized at a higher plane-wave cut-off of 700 eV with lattice constants 20.09, 19.74, and 13.14 Å. The entire unit cell was fully relaxed (no constraint on atom positions) during the optimization. Structures failing to converge after 500 ionic steps were excluded from the fitting. The relative energy (ΔE) for the $Cr^{6+}OO$ structures shown in Figure 4c was calculated as:

$$\Delta E = E(Cr^{6+}OO)_x - E(SiMFI)_x$$

Where x represents each of the 12 crystallographically unique T-sites in MFI. The 12 open defect sites were geometrically optimized by DFT following the same setups. This energy was then normalized by the lowest energy to allow a more straightforward comparison.

2.3 Theoretical EXAFS Fitting

The theoretical EXAFS fittings were performed using the QuantEXAFS package[12] and the Larch library[13] as the back end. By taking the DFT-optimized structures "as-is" to feed into the fitting algorithms, the unique local environment of Cr species with fixed coordination numbers provided by DFT could be captured. In the current analysis, ten fitting parameters were used to fit the experimental EXAFS spectra, which are within the upper bound of 10.3 independent variables based on the Nyquist criterion. The ten fitting parameters include (1) A single energy correction term ΔE_0 for all relevant scattering paths; (2) Three distance correction terms ΔR, categorizing scattering paths by effective scattering distances (R_{eff}), using different values for each of the three main shells in ranges < 1.7 Å, 1.7 to 2.2 Å, and > 2.2 Å in the *R*-space; (3) Six disorder terms σ^2 classifying paths based on both R_{eff} and bond types, which includes single scattered Cr=O (σ^2_0) and single scattered Cr-O (σ^2_1) for the first shell, single scattered Cr-O (σ^2_2) and single scattered Cr-Si (σ^2_3) for the second shell, and additional parameters for all other single scattering paths (σ^2_4) and all the multiple scattering paths (σ^2_5).

The passive electronic reduction term S_0^2 was 0.85, determined by fitting EXAFS spectra of Cr foil. All fittings were done in the *R*-space of k^2-weighted EXAFS, with *k*-range 3.7–9.3 A^{-1} and *R*-range 0.6–3.2 Å.

3. Supporting Tables and Figures

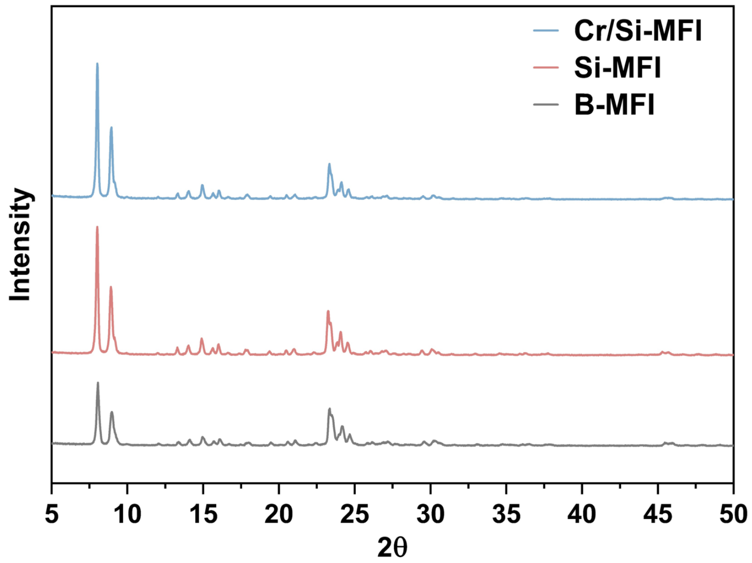

Figure S1. XRD of MFI samples. X-ray diffraction patterns of B-MFI (grey), Si-MFI (pink), and Cr/Si-MFI (blue).

Table S1. Elemental composition of samples determined by ICP.

Sample	wt% B	wt% Cr
B-MFI	0.513	-
Si-MFI	0.111	-
Cr/Si-MFI	0.111	0.547

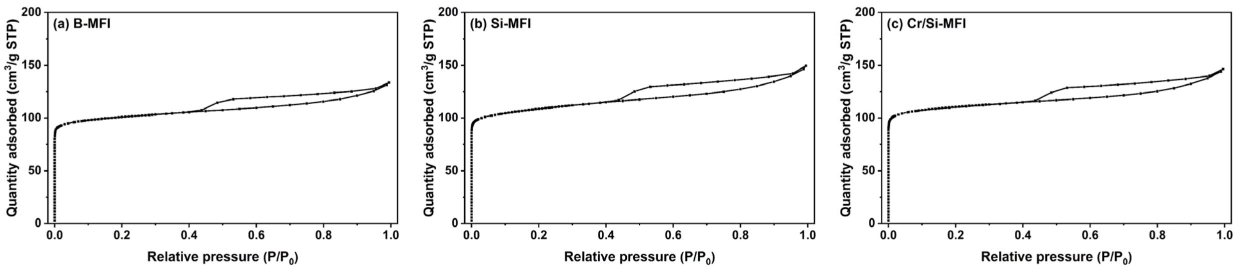

Figure S2. N_2 physisorption isotherms. N_2 physisorption isotherms collected at 77 K.

Table S2. N_2 physisorption results.

Sample	BET surface area (m^2/g)	Micropore surface area (m^2/g)[a]	External surface area (m^2/g)[a]	Micropore volume (cm^3/g)[a]
B-MFI	398.2	333.1	65.1	0.128
Si-MFI	423.4	334.5	88.9	0.133
Cr/Si-MFI	437.6	373.0	64.6	0.143

[a]Determined by t-plot method. All samples were degassed under vacuum at 350 °C for 8 hours before N_2 adsorption.

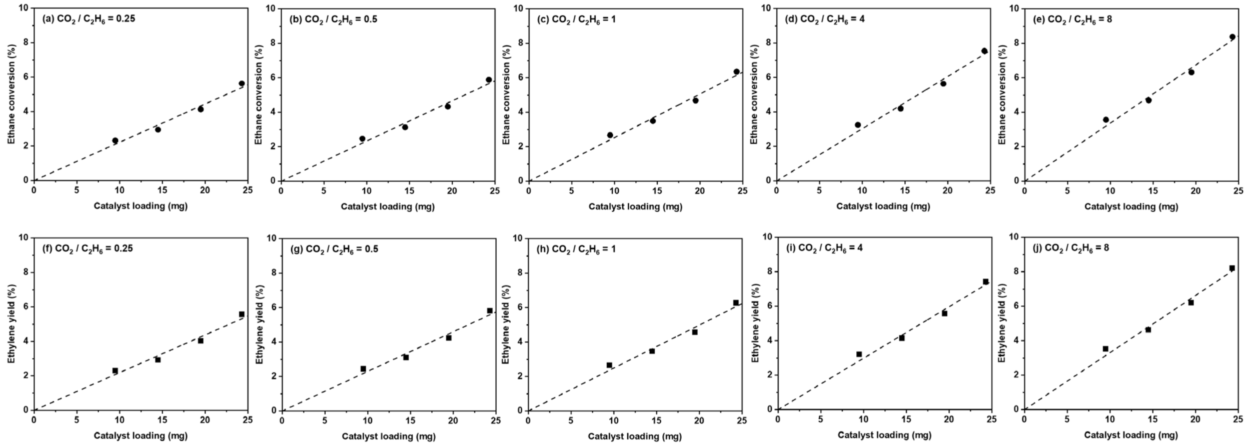

Figure S3. Ethane conversion and ethylene yield measured in the kinetic-controlled regime. (a-e) Ethane conversion as a function of catalyst loading over 0.5 wt% Cr/Si-MFI. (f-j) Ethylene yield as a function of catalyst loading over 0.5 wt% Cr/Si-MFI. Reaction condition: T = 650 °C; P = 2.5 psig; total flow rate: 48 sccm; C_2H_6 flow rate: 4 sccm; CO_2 and N_2 flow rates set based on the targeted CO_2 / C_2H_6 mole ratio; catalyst amount: 8–25 mg.

Table S3. Ethane conversion summary of blank reactor tests.

**Temperature (°C)**	**Ethane conversion of 4 g α-alumina (%)**	**Ethane conversion per mg Si-MFI (%)**
610	0.012	0.001
630	0.014	0.001
650	0.024	0.003
670	0.056	0.006
690	0.148	0.014
710	0.346	0.031

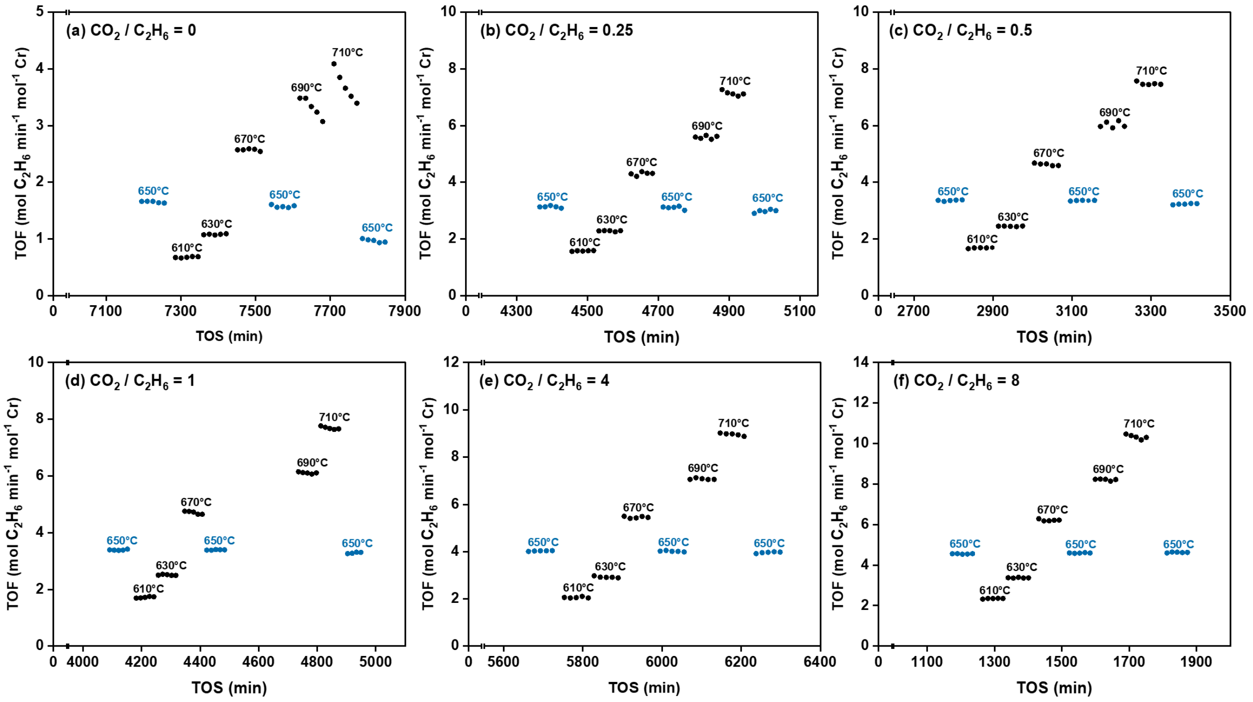

Figure S4. Time-on-stream rate of ethane conversion with different CO_2 / C_2H_6 mole ratio conditions. Cr/Si-MFI only deactivated at high temperature conditions (above 670 °C) without cofed CO_2. Reaction condition: T = 610–710 °C; P = 2.5 psig; total flow rate: 48 sccm; C_2H_6 flow rate: 4 sccm; CO_2 and N_2 flow rates set based on the targeted CO_2 / C_2H_6 mole ratio; catalyst amount: 20–25 mg.

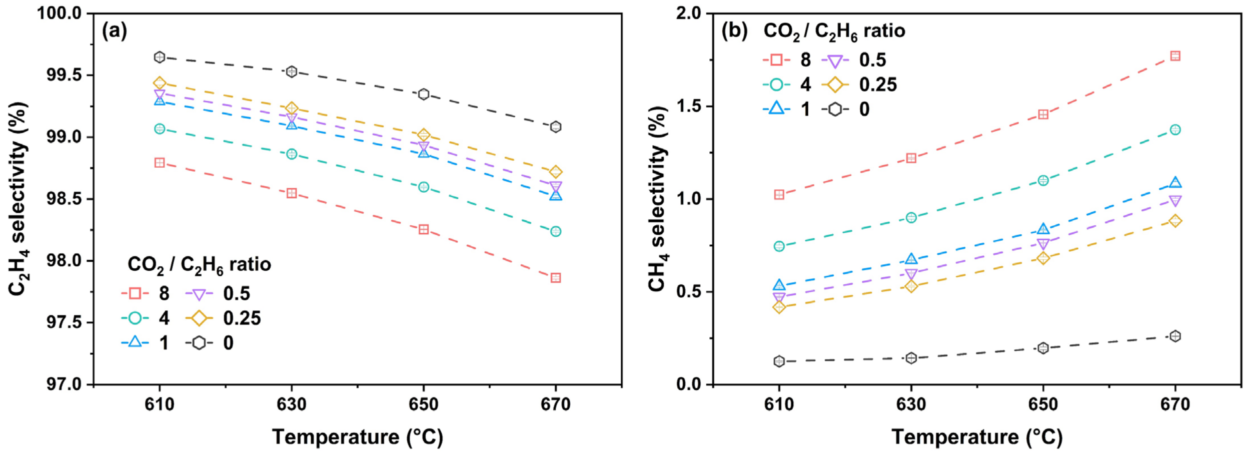

Figure S5. Product distribution of ethane dehydrogenation over Cr/Si-MFI. (a) Ethylene selectivity as a function of temperature with varying CO_2 / C_2H_6 feed ratios. (b) Methane selectivity as a function of temperature with varying CO_2 / C_2H_6 feed ratios. The reported selectivity was calculated by averaging five data points collected during the initial 75 min steady-state time on stream. Reaction condition: T = 610–670 °C; P = 2.5 psig; total flow rate: 48 sccm; C_2H_6 flow rate: 4 sccm; CO_2 and N_2 flow rates set based on the targeted CO_2 / C_2H_6 mole ratio; catalyst amount: 20–25 mg.

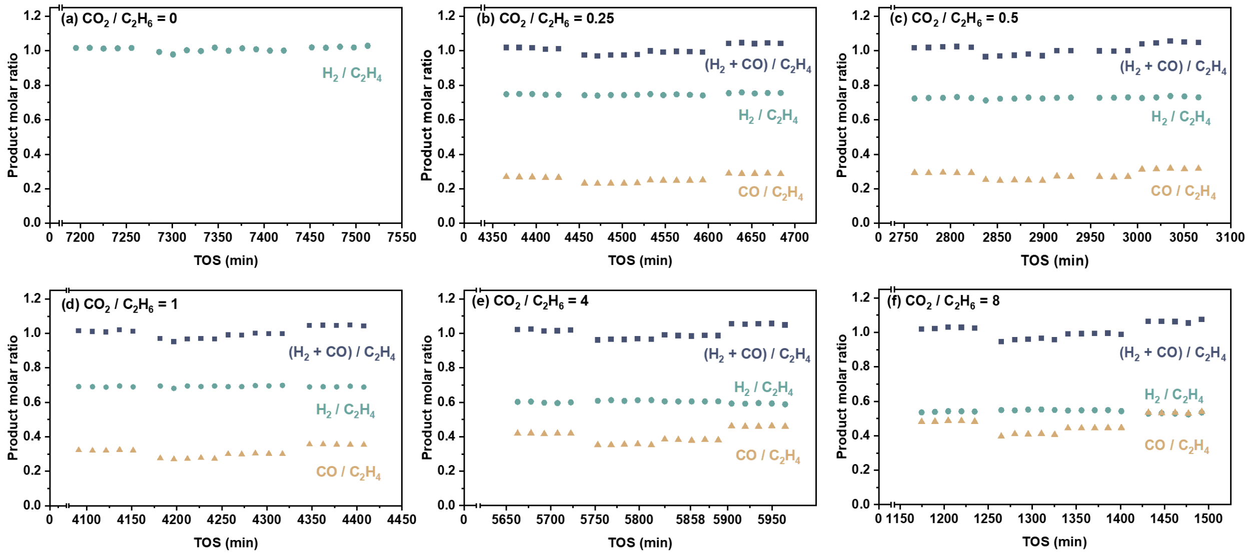

Figure S6. Time-on-stream product distribution in different CO_2 / C_2H_6 feed conditions.

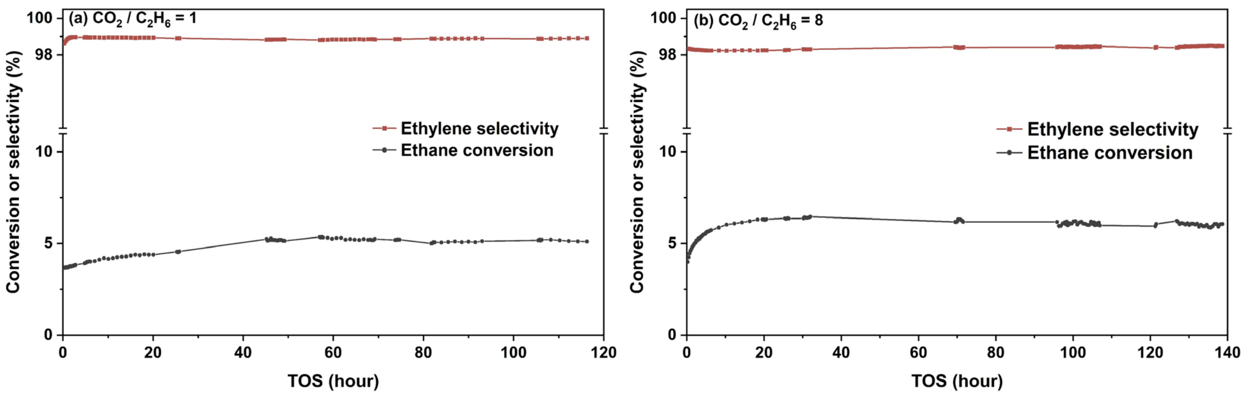

Figure S7. Time-on-stream ethane conversion and ethylene selectivity over 120 h, indicating good stability of Cr/Si-MFI during CO_2-EDH reaction. (a) Stability test under feed condition CO_2 / C_2H_6 = 1. (b) Stability test under feed condition CO_2 / C_2H_6 = 8. Reaction condition: T = 650 °C; P = 2.5 psig; total flow rate: 48 sccm; C_2H_6 flow rate: 4 sccm; CO_2 and N_2 flow rates set based on the targeted CO_2 / C_2H_6 mole ratio; catalyst amount: 20–25 mg.

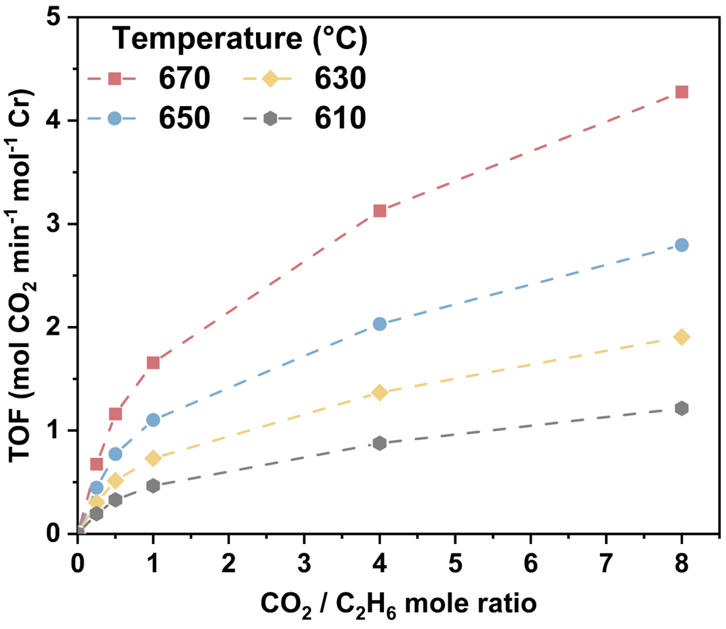

Figure S8. Rate of CO_2 conversion per Cr site vs. CO_2 / C_2H_6 mole ratio at four temperatures. Reaction condition: T = 610–670 °C; P = 2.5 psig; total flow rate: 48 sccm; C_2H_6 flow rate: 4 sccm; CO_2 and N_2 flow rates set based on the targeted CO_2 / C_2H_6 mole ratio; catalyst amount: 20–25 mg.

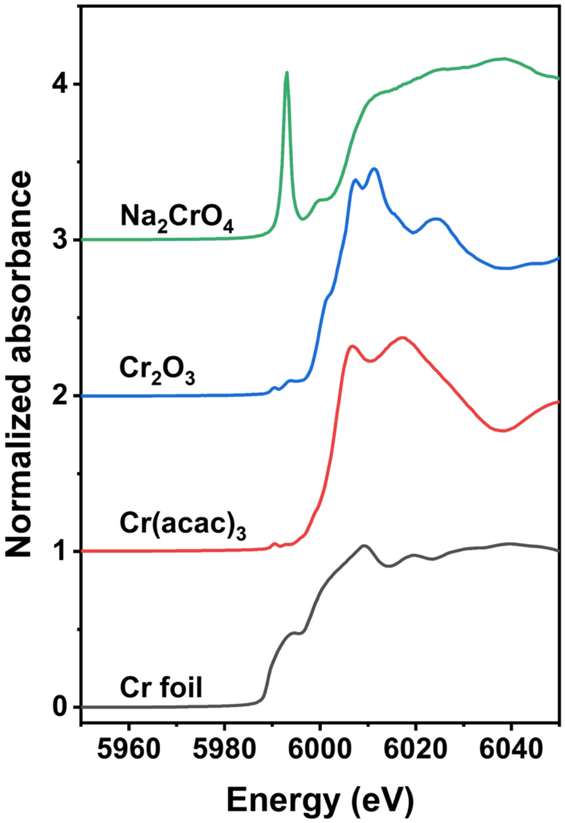

Figure S9. XANES characterizing Cr reference compounds. Spectra were recorded in ambient air at room temperature.

Table S4. Theoretical EXAFS fit parameters – the most stable $Cr^{6+}OO$ model.

Category	N	R [Å]	σ^2 [Å^2]	ΔE_0 [eV]	r-factor	Reduced χ^2
First shell Cr=O	2	1.60 ± 0.00	0.010	-10	0.016	11.9
First shell Cr-O(-Si)	2	1.76 ± 0.01	0.015			
Second shell Cr-O(-H)	1	2.85 ± 0.00	0.050			
Second shell Cr-Si	2	3.17 ± 0.02	0.018			
All other single scattering			0.027			
Multiple scattering			0.050			

Notation: N, coordination number; R, scattering path length; σ^2, disorder term; ΔE_0, energy correction factor.

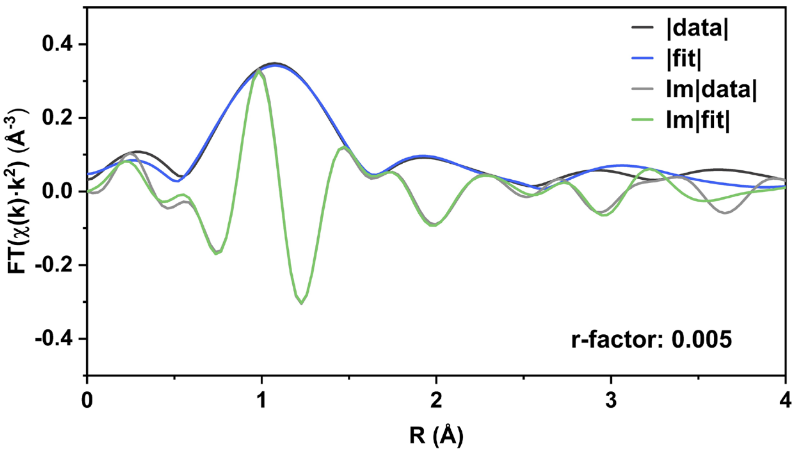

Figure S10. Theoretical EXAFS fitting of the best-fit Cr^{6+}OO structural model (labeled in Figure 4c with an r-factor of 0.005). Magnitudes (fit, blue; experiment, black) and imaginary parts (fit, green; experiment, gray) of Fourier-transformed EXAFS of calcined Cr/Si-MFI.

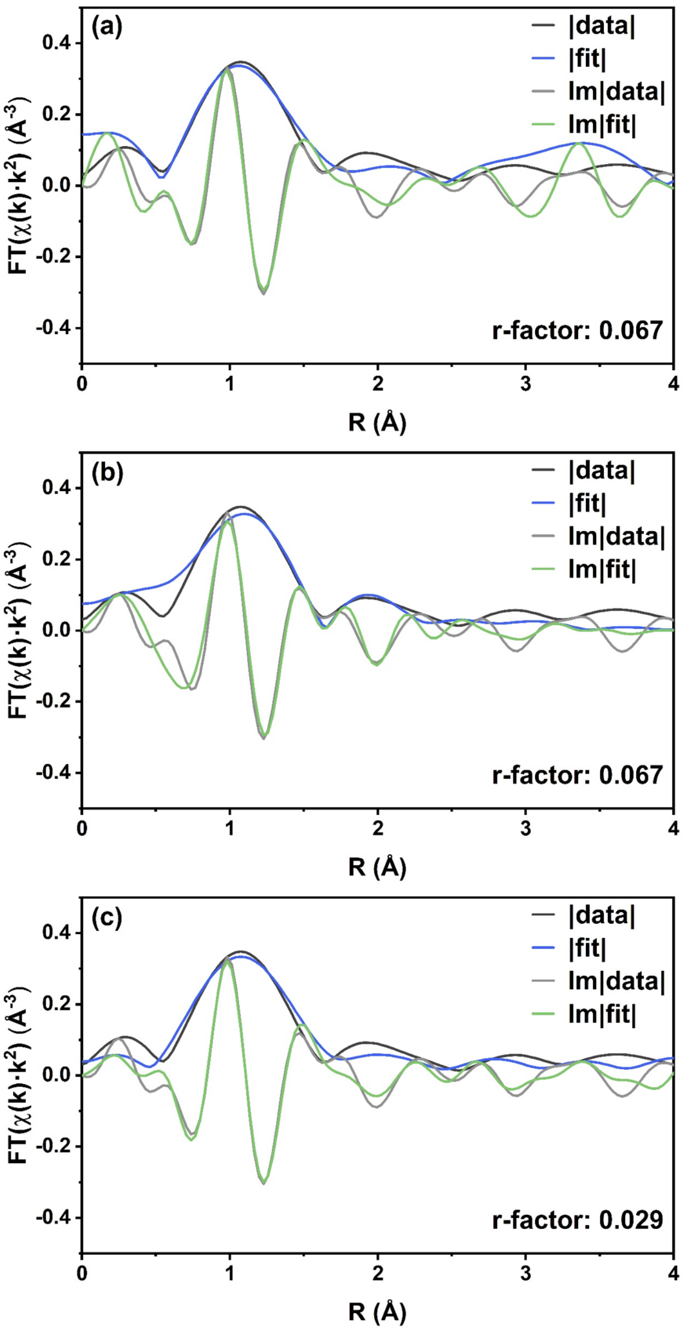

Figure S11. Theoretical EXAFS fitting of the best-fit (a) Cr^{2+} (b) Cr^{3+} (c) $Cr^{6+}O$ structural models. Magnitudes (fit, blue; experiment, black) and imaginary parts (fit, green; experiment, gray) of Fourier-transformed EXAFS of calcined Cr/Si-MFI.

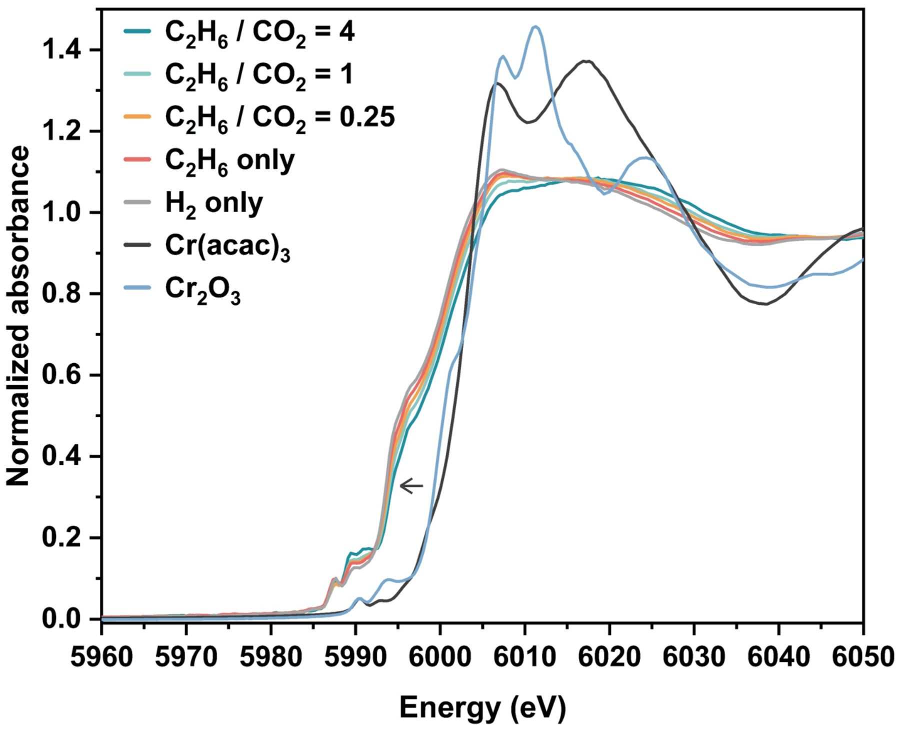

Figure S12. Comparison with Cr(III) reference compounds in XANES. Cr/Si-MFI *operando* spectra were recorded when sample was exposed to flowing CO_2 / C_2H_6 / He mixture or flowing H_2 at 650 °C. Cr(III) reference compounds spectra were recorded in ambient air at room temperature.

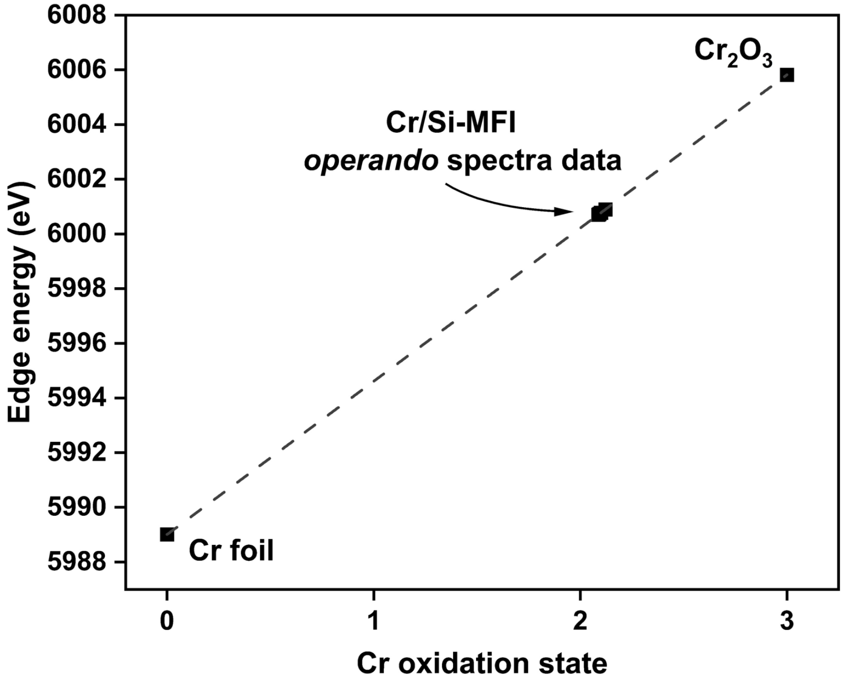

Figure S13. Edge position comparison between Cr reference compounds and Cr/Si-MFI sample under different reactive conditions. Cr reference compounds spectra were recorded in ambient air at room temperature. Cr/Si-MFI *operando* spectra were recorded when sample was exposed to flowing CO_2 / C_2H_6 / He mixture or flowing H_2 at 650 °C.

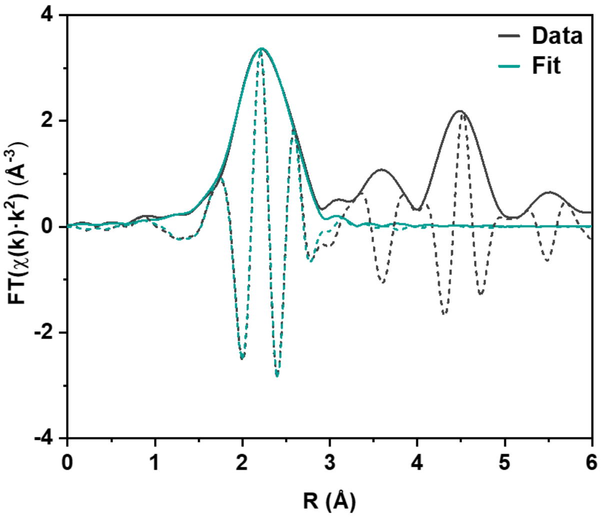

Figure S14. EXAFS fitting of spectrum characterizing Cr foil. Magnitude (solid) and imaginary part (dash) of the k^2-weighted Fourier-transform of EXAFS characterizing Cr foil (dark grey) compared to the best-fit model (green). Coordination number N was fixed to bulk values in this model.

Table S5. Best-fit EXAFS model – Cr foil, used to determine the S_0^2 parameter.

Scattering path [R_{eff} (Å)]	N	S_0^2	ΔE_0 [eV]	R [Å]	$10^3 \cdot \sigma^2$ [Å^2]	r-factor
Cr-Cr [2.49]	8	0.85 ± 0.17	2.8 ± 2.2	2.49 ± 0.01	7.3 ± 1.7	0.008
Cr-Cr [2.87]	6			2.85 ± 0.01	4.5 ± 1.7	

Notation: N, coordination number; S_0^2, passive electronic reduction factor; ΔE_0, energy correction factor; R, scattering path length; σ^2, disorder term.

k-range: 3.0–12.0 Å^{-1}

R-range: 1.0–3.1 Å

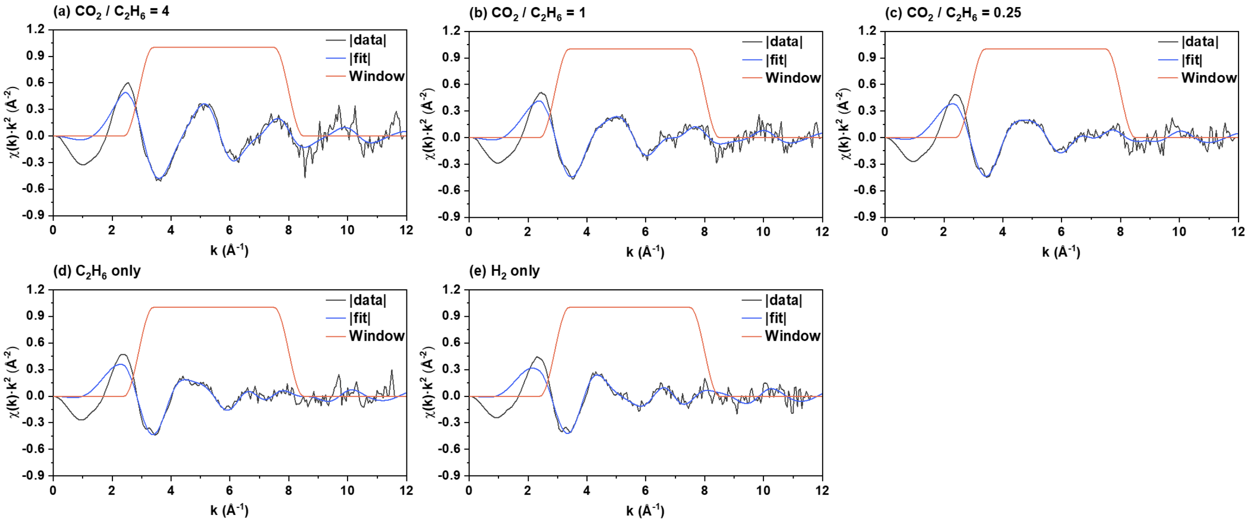

Figure S15. EXAFS k-space fitting of spectra characterizing Cr/Si-MFI with k-range of 2.9–8.0 Å^{-1}. The best fit model contains Cr-O [1.79 Å], Cr-O [1.99 Å] and Cr-Si [3.18 Å] paths. (fit, blue; experiment, black; red, window).

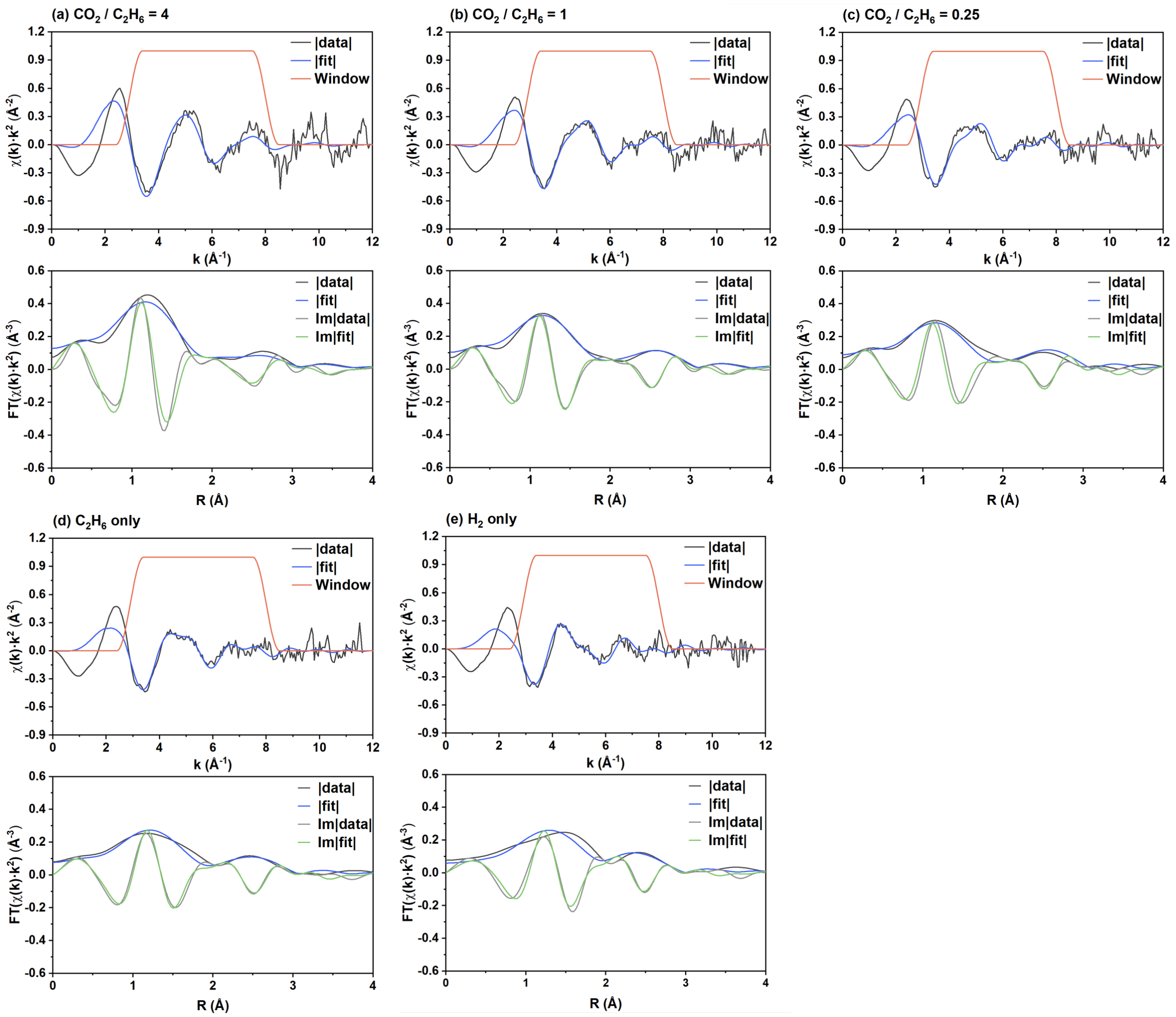

Figure S16. EXAFS fitting of rejected model containing Cr-O [1.99 Å] and Cr-Si [3.18 Å] paths. Top: EXAFS spectra in *k*-space (fit, blue; experiment, black; red, window); Bottom: magnitude (fit, blue; experiment, black) and imaginary parts (fit, green; experiment, gray) of k^2-weighted, Fourier-transformed EXAFS of Cr/Si-MFI.

Table S6. Rejected EXAFS model containing Cr-O [1.99 Å] and Cr-Si [3.18 Å] paths [a,b].

Condition	Scattering path [R_{eff} (Å)]	N	R [Å]	ΔE_0 [eV][c]	$10^3 \cdot \sigma^2$ [$Å^2$][d]	S_0^2	r-factor
$CO_2/C_2H_6 = 4$	Cr-O [1.99]	2.76 ± 0.99	1.77 ± 0.03	-9.54 ± 4.09	17.07 ± 5.78	0.85[e]	0.07
	Cr-Si [3.18]	0.51 ± 0.68	3.18 ± 0.08	-0.04 ± 6.72	8.79 ± 12.15		
$CO_2/C_2H_6 = 1$	Cr-O [1.99]	2.17 ± 0.82	1.77 ± 0.03	-9.54 ± 4.09	17.07 ± 5.78		
	Cr-Si [3.18]	0.91 ± 0.89	3.18 ± 0.08	-0.04 ± 6.72	8.79 ± 12.15		
$CO_2/C_2H_6 = 0.25$	Cr-O [1.99]	1.87 ± 0.71	1.77 ± 0.03	-9.54 ± 4.09	17.07 ± 5.78		
	Cr-Si [3.18]	1.02 ± 0.91	3.18 ± 0.08	-0.04 ± 6.72	8.79 ± 12.15		
C_2H_6 only	Cr-O [1.99]	1.89 ± 0.71	1.83 ± 0.03	-9.54 ± 4.09	17.07 ± 5.78		
	Cr-Si [3.18]	0.87 ± 0.85	3.18 ± 0.08	-0.04 ± 6.72	8.79 ± 12.15		
H_2 only	Cr-O [1.99]	1.87 ± 0.74	1.88 ± 0.04	-9.54 ± 4.09	17.07 ± 5.78		
	Cr-Si [3.18]	0.86 ± 0.88	3.13 ± 0.10	-0.04 ± 6.72	8.79 ± 12.15		

[a]k-range of 2.9–8.0 $Å^{-1}$ and R-range of 1.0–3.1 Å were chosen for the simultaneous fittings based on rejected EXAFS model containing Cr-O [1.99 Å] and Cr-Si [3.18 Å] paths.

[b]Notation: N, coordination number; R, scattering path length; ΔE_0, energy correction factor; σ^2, disorder term; S_0^2, passive electronic reduction factor.

[c]ΔE_0 for Cr-O [1.99] scattering path of each spectrum were constrained to be equal, with the same applied to Cr-Si [3.18] scattering path.

[d]σ^2 for the Cr-O [1.99] scattering path of each spectrum were constrained to be equal, with the same applied to Cr-Si [3.18] scattering path.

[e]S_0^2 was set as 0.85 based on the modeling of the Cr foil EXAFS spectrum (Figure S14 and Table S5).

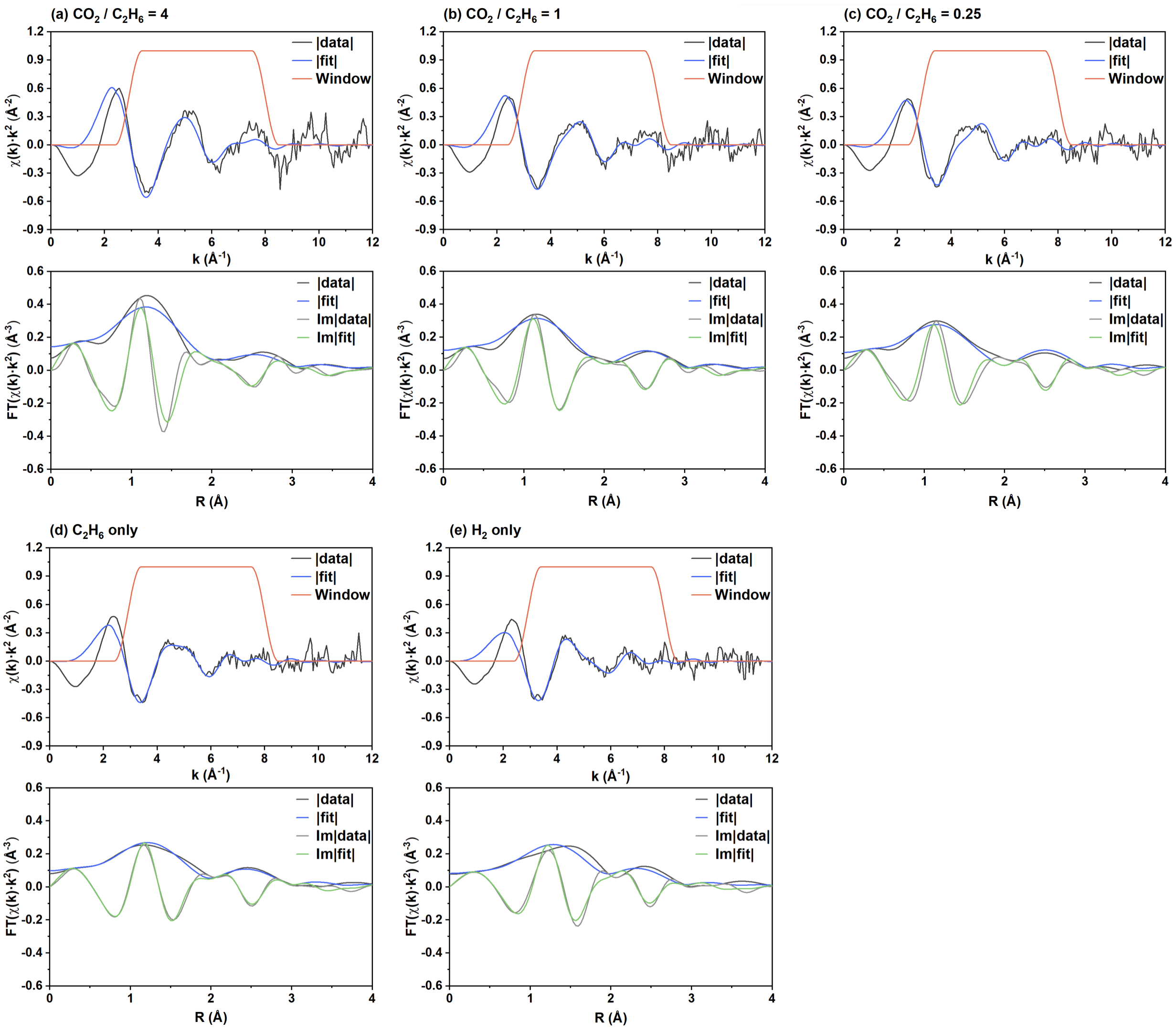

Figure S17. EXAFS fitting of rejected model containing Cr-O [1.79 Å] and Cr-Si [3.18 Å] paths. Top: EXAFS spectra in *k*-space (fit, blue; experiment, black; red, window); Bottom: magnitude (fit, blue; experiment, black) and imaginary parts (fit, green; experiment, gray) of k^2-weighted, Fourier-transformed EXAFS of Cr/Si-MFI.

Table S7. Rejected EXAFS model containing Cr-O [1.79 Å] and Cr-Si [3.18 Å] paths [a,b].

Condition	Scattering path [R_{eff} (Å)]	N	R [Å]	ΔE_0 [eV][c]	$10^3 \cdot \sigma^2$ [Å^2][d]	S_0^2	r-factor
$CO_2/C_2H_6 = 4$	Cr-O [1.79]	4.46 ± 0.63	1.80 ± 0.02	-6.88 ± 2.74	23.95	0.85[e]	0.07
	Cr-Si [3.18]	0.75 ± 0.57	3.11 ± 0.04		9.73		
$CO_2/C_2H_6 = 1$	Cr-O [1.79]	3.66 ± 0.64	1.80 ± 0.02		23.95		
	Cr-Si [3.18]	1.15 ± 0.60	3.11 ± 0.04		9.73		
$CO_2/C_2H_6 = 0.25$	Cr-O [1.79]	3.23 ± 0.56	1.80 ± 0.02		23.95		
	Cr-Si [3.18]	1.26 ± 0.50	3.11 ± 0.04		9.73		
C_2H_6 only	Cr-O [1.79]	3.18 ± 0.56	1.85 ± 0.03		23.95		
	Cr-Si [3.18]	0.93 ± 0.57	3.11 ± 0.04		9.73		
H_2 only	Cr-O [1.79]	3.07 ± 0.67	1.89 ± 0.03		23.95		
	Cr-Si [3.18]	0.77 ± 0.65	3.07 ± 0.08		9.73		

[a] k-range of 2.9–8.0 Å^{-1} and R-range of 1.0–3.1 Å were chosen for the simultaneous fittings based on rejected EXAFS model containing Cr-O [1.79 Å] and Cr-Si [3.18 Å] paths.

[b] Notation: N, coordination number; R, scattering path length; ΔE_0, energy correction factor; σ^2, disorder term; S_0^2, passive electronic reduction factor.

[c] ΔE_0 for Cr-O [1.79] scattering path of each spectrum were constrained to be equal, with the same applied to Cr-Si [3.18] scattering path.

[d] σ^2 for the Cr-O [1.79] scattering path of each spectrum were constrained to be equal, with the same applied to Cr-Si [3.18] scattering path. All σ^2 terms were kept constant at the value of the first fit to decrease statistical error.

[e] S_0^2 was set as 0.85 based on the modeling of the Cr foil EXAFS spectrum (Figure S14 and Table S5).

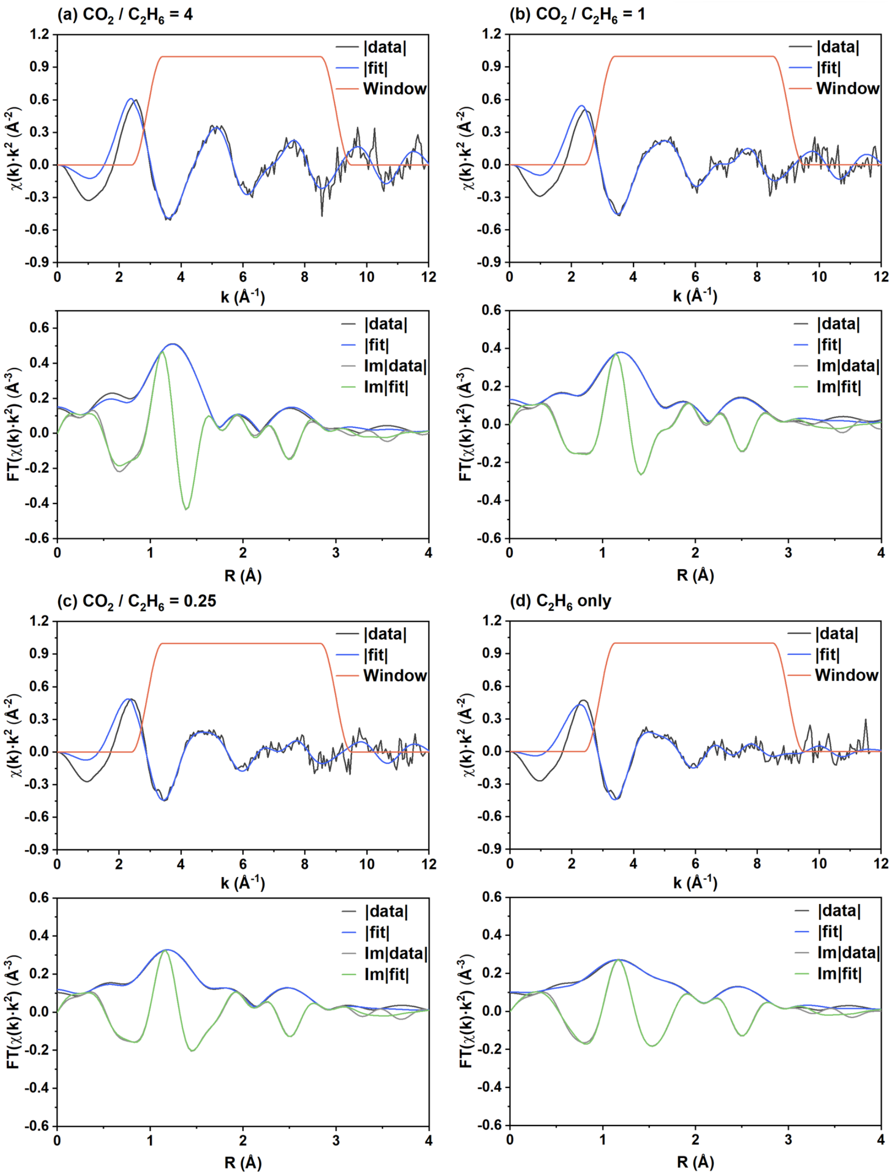

Figure S18. EXAFS fitting of rejected model containing Cr-O [1.79 Å], Cr-O [1.99 Å], Cr-Si [3.18 Å] and extra Cr-C [2.07 Å] paths. Top: EXAFS spectra in *k*-space (fit, blue; experiment, black; red, window); Bottom: magnitude (fit, blue; experiment, black) and imaginary parts (fit, green; experiment, gray) of k^2-weighted, Fourier-transformed EXAFS of Cr/Si-MFI.

Table S8. Rejected EXAFS model containing Cr-O [1.79 Å], Cr-O [1.99 Å], Cr-Si [3.18 Å] and extra Cr-C [2.07 Å] paths[a,b].

Condition	Scattering path [R_{eff} (Å)]	N	R [Å]	ΔE_0 [eV][c]	$10^3 \cdot \sigma^2$ [Å^2][d]	S_0^2	r-factor
$CO_2/C_2H_6 = 4$	Cr-O [1.79]	4.06 ± 2.38	1.87 ± 0.05	-5.44 ± 0.82	13.11	0.85[e]	0.002
	Cr-O [1.99]	-3.96 ± 2.17	1.95± 0.02	-5.98 ± 1.72	6.42		
	Cr-C [2.07]	2.28 ± 0.65	2.08 ± 0.02	-1.51 ± 2.06	3.88		
	Cr-Si [3.18]	1.25 ± 0.14	3.11 ± 0.01	-5.44 ± 0.82	8.33		
$CO_2/C_2H_6 = 1$	Cr-O [1.79]	3.25 ± 1.80	1.87 ± 0.05	-5.44 ± 0.82	13.11		
	Cr-O [1.99]	-2.80 ± 1.78	1.95± 0.02	-5.98 ± 1.72	6.42		
	Cr-C [2.07]	2.04 ± 0.52	2.08 ± 0.02	-1.51 ± 2.06	3.88		
	Cr-Si [3.18]	1.12 ± 0.12	3.11 ± 0.01	-5.44 ± 0.82	8.33		
$CO_2/C_2H_6 = 0.25$	Cr-O [1.79]	2.77 ± 1.47	1.87 ± 0.05	-5.44 ± 0.82	13.11		
	Cr-O [1.99]	-2.04 ± 1.54	1.95± 0.02	-5.98 ± 1.72	6.42		
	Cr-C [2.07]	1.71 ± 0.45	2.08 ± 0.02	-1.51 ± 2.06	3.88		
	Cr-Si [3.18]	0.99 ± 0.11	3.11 ± 0.01	-5.44 ± 0.82	8.33		
C_2H_6 only	Cr-O [1.79]	1.66 ± 0.28	1.80 ± 0.01	-5.44 ± 0.82	13.11		
	Cr-O [1.99]	0.16 ± 0.55	2.05 ± 0.28	-5.98 ± 1.72	6.42		
	Cr-C [2.07]	0.72 ± 0.77	2.08 ± 0.02	-1.51 ± 2.06	3.88		
	Cr-Si [3.18]	0.94 ± 0.10	3.11 ± 0.01	-5.44 ± 0.82	8.33		

[a]*k*-range of 2.9–9.0 Å^{-1} and *R*-range of 1.0–3.1 Å were chosen for the simultaneous fittings based on the rejected model containing Cr-O [1.79 Å], Cr-O [1.99 Å], Cr-Si [3.18 Å] and extra Cr-C [2.07 Å] paths. Cr-C scattering path was obtained from a $Cr_{23}C_6$ cif file (mp-723, from the Materials Project[4]) with R_{eff} of 2.07 Å.

[b]Notation: N, coordination number; R, scattering path length; ΔE_0, energy correction factor; σ^2, disorder term; S_0^2, passive electronic reduction factor.

[c]ΔE_0 for Cr-O [1.79] and Cr-Si [3.18] scattering paths were constrained to be equal.

[d]σ^2 for the Cr-O [1.79] scattering path of each spectrum were constrained to be equal, with the same applied for Cr-O [1.99], Cr-C [2.07] and Cr-Si [3.18] scattering paths.

[e]S_0^2 was set as 0.85 based on the modeling of the Cr foil EXAFS spectrum (Figure S14 and Table S5).

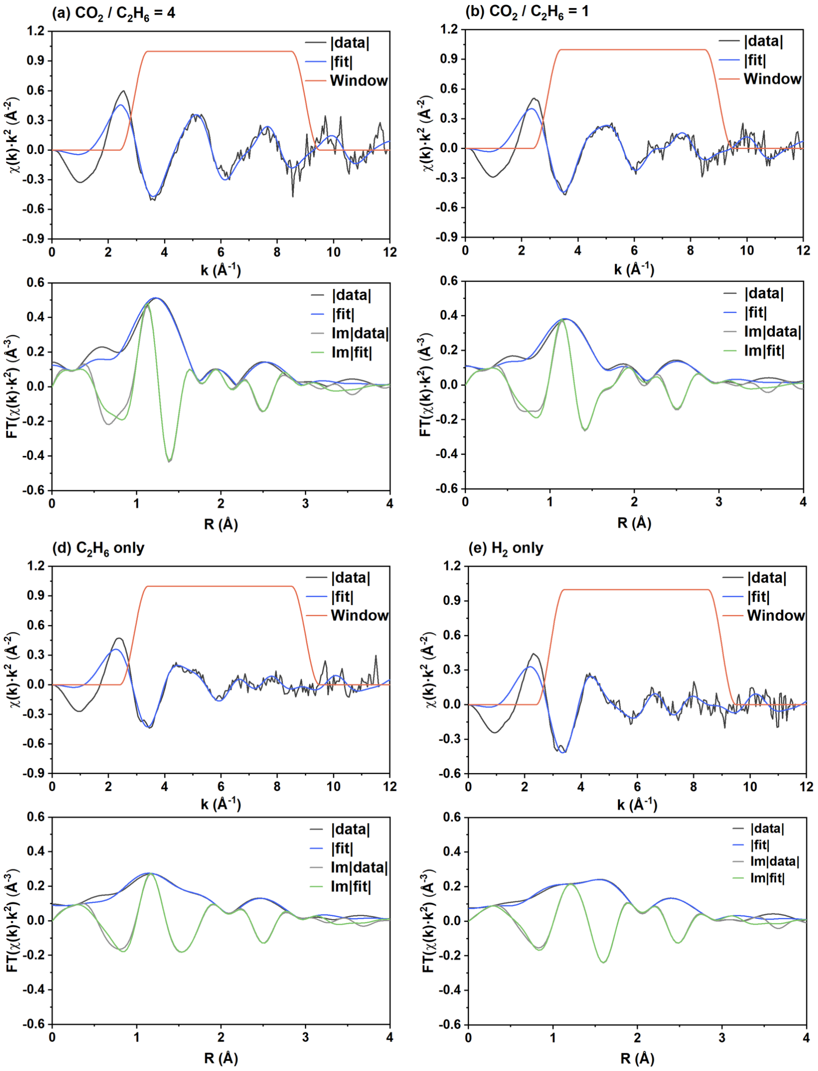

Figure S19. EXAFS fitting of spectra characterizing Cr/Si-MFI with *k*-range of 2.9–9.0 Å^{-1}. Top: EXAFS spectra in *k*-space (fit, blue; experiment, black; red, window); Bottom: magnitude (fit, blue; experiment, black) and imaginary parts (fit, green; experiment, gray) of k^2-weighted, Fourier-transformed EXAFS of Cr/Si-MFI.

Table S9. Best-fit EXAFS model – *k*-range of 2.9–9.0 Å^{-1}[a,b].

Condition	Scattering path [R_{eff} (Å)]	N	R [Å]	ΔE_0 [eV][c]	$10^3\cdot\sigma^2$ [Å^2][d]	S_0^2	r-factor
CO_2/C_2H_6 = 4	Cr-O [1.79]	1.41 ± 0.05	1.76 ± 0.01	-4.32 ± 1.13	2.8	0.85[e]	0.004
	Cr-O [1.99]	0.46 ± 0.14	1.97 ± 0.01	-3.79 ± 2.08	6.9		
	Cr-Si [3.18]	0.72 ± 0.10	3.12 ± 0.01	-4.32 ± 1.13	4.4		
CO_2/C_2H_6 = 1	Cr-O [1.79]	1.10 ± 0.08	1.76 ± 0.01	-4.32 ± 1.13	2.8		
	Cr-O [1.99]	0.86 ± 0.15	1.97 ± 0.01	-3.79 ± 2.08	6.9		
	Cr-Si [3.18]	0.68 ± 0.09	3.12 ± 0.01	-4.32 ± 1.13	4.4		
CO_2/C_2H_6 = 0.25	Cr-O [1.79]	0.93 ± 0.09	1.76 ± 0.01	-4.32 ± 1.13	2.8		
	Cr-O [1.99]	1.07 ± 0.15	1.97 ± 0.01	-3.79 ± 2.08	6.9		
	Cr-Si [3.18]	0.59 ± 0.08	3.12 ± 0.01	-4.32 ± 1.13	4.4		
C_2H_6 only	Cr-O [1.79]	0.74 ± 0.08	1.75 ± 0.01	-4.32 ± 1.13	2.8		
	Cr-O [1.99]	1.21 ± 0.17	1.96 ± 0.01	-3.79 ± 2.08	6.9		
	Cr-Si [3.18]	0.64 ± 0.06	3.12 ± 0.01	-4.32 ± 1.13	4.4		
H_2 only	Cr-O [1.79]	0.54 ± 0.10	1.73 ± 0.01	-4.32 ± 1.13	2.8		
	Cr-O [1.99]	1.42 ± 0.18	1.96 ± 0.01	-3.79 ± 2.08	6.9		
	Cr-Si [3.18]	0.58 ± 0.08	3.10 ± 0.01	-4.32 ± 1.13	4.4		

[a]*k*-range of 2.9–9.0 Å^{-1} and *R*-range of 1.0–3.1 Å were chosen for the simultaneous fittings based on the best-fit model.

[b]Notation: N, coordination number; R, scattering path length; ΔE_0, energy correction factor; σ^2, disorder term; S_0^2, passive electronic reduction factor.

[c]ΔE_0 for Cr-O [1.79] and Cr-Si [3.18] scattering paths were constrained to be equal.

[d]σ^2 for the Cr-O [1.79] scattering path of each spectrum were constrained to be equal, with the same applied for Cr-O [1.99] and Cr-Si [3.18] scattering paths. All σ^2 terms were kept constant at the value of the first fit to decrease statistical error.

[e]S_0^2 was set as 0.85 based on the modeling of the Cr foil EXAFS spectrum (Figure S14 and Table S5).

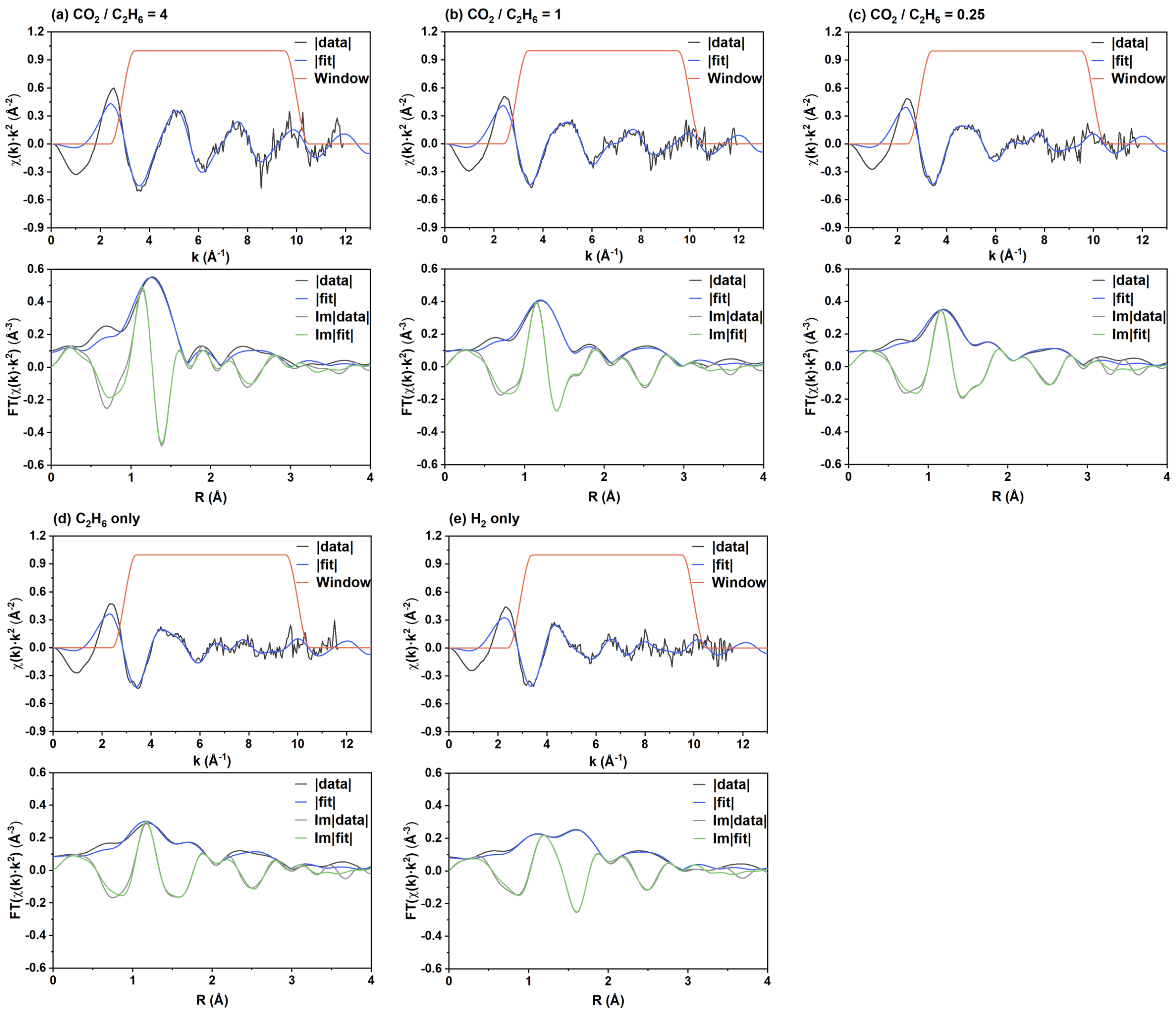

Figure S20. EXAFS fitting of spectra characterizing Cr/Si-MFI with *k*-range of 2.9–10.0 Å^{-1}. Top: EXAFS spectra in *k*-space (fit, blue; experiment, black; red, window); Bottom: magnitude (fit, blue; experiment, black) and imaginary parts (fit, green; experiment, gray) of k^2-weighted, Fourier-transformed EXAFS of Cr/Si-MFI.

Table S10. Best-fit EXAFS model – k-range of 2.9–10.0 Å^{-1}[a,b].

Condition	Scattering path [R_{eff} (Å)]	N	R [Å]	ΔE_0 [eV][c]	$10^3 \cdot \sigma^2$ [Å^2][d]	S_0^2	r-factor
$CO_2/C_2H_6 = 4$	Cr-O [1.79]	1.30 ± 0.17	1.76 ± 0.01	-5.00 ± 1.62	2.0	0.85[e]	0.009
	Cr-O [1.99]	0.27 ± 0.21	1.98 ± 0.02	-1.07 ± 2.48	4.9		
	Cr-Si [3.18]	0.78 ± 0.32	3.12 ± 0.02	-5.00 ± 1.62	6.7		
$CO_2/C_2H_6 = 1$	Cr-O [1.79]	1.01 ± 0.16	1.76 ± 0.01	-5.00 ± 1.62	2.0		
	Cr-O [1.99]	0.65 ± 0.17	1.98 ± 0.02	-1.07 ± 2.48	4.9		
	Cr-Si [3.18]	0.80 ± 0.18	3.12 ± 0.02	-5.00 ± 1.62	6.7		
$CO_2/C_2H_6 = 0.25$	Cr-O [1.79]	0.85 ± 0.16	1.76 ± 0.01	-5.00 ± 1.62	2.0		
	Cr-O [1.99]	0.83 ± 0.17	1.98 ± 0.02	-1.07 ± 2.48	4.9		
	Cr-Si [3.18]	0.73 ± 0.16	3.12 ± 0.02	-5.00 ± 1.62	6.7		
C_2H_6 only	Cr-O [1.79]	0.72 ± 0.14	1.76 ± 0.01	-5.00 ± 1.62	2.0		
	Cr-O [1.99]	0.90 ± 0.19	1.99 ± 0.02	-1.07 ± 2.48	4.9		
	Cr-Si [3.18]	0.73 ± 0.15	3.12 ± 0.02	-5.00 ± 1.62	6.7		
H_2 only	Cr-O [1.79]	0.47 ± 0.12	1.75 ± 0.02	-5.00 ± 1.62	2.0		
	Cr-O [1.99]	1.09 ± 0.21	1.98 ± 0.02	-1.07 ± 2.48	4.9		
	Cr-Si [3.18]	0.67 ± 0.15	3.09 ± 0.02	-5.00 ± 1.62	6.7		

[a] k-range of 2.9–10.0 Å^{-1} and R-range of 1.0–3.1 Å were chosen for the simultaneous fittings based on the best-fit model.

[b] Notation: N, coordination number; R, scattering path length; ΔE_0, energy correction factor; σ^2, disorder term; S_0^2, passive electronic reduction factor.

[c] ΔE_0 for Cr-O [1.79] and Cr-Si [3.18] scattering paths were constrained to be equal.

[d] σ^2 for the Cr-O [1.79] scattering path of each spectrum were constrained to be equal, with the same applied for Cr-O [1.99] and Cr-Si [3.18] scattering paths. All σ^2 terms were kept constant at the value of the first fit to decrease statistical error.

[e] S_0^2 was set as 0.85 based on the modeling of the Cr foil EXAFS spectrum (Figure S14 and Table S5).

4. References

(1) Forni, L.; Fornasari, G.; Trifirò, F.; Aloise, A.; Katovic, A.; Giordano, G.; Nagy, J. B. Calcination and Deboronation of B-MFI Applied to the Vapour Phase Beckmann Rearrangement. *Microporous Mesoporous Mater*. **2007**, *101*(1-2), 161-168. DOI: 10.1016/j.micromeso.2006.12.013

(2) Felvey, N. W.; Meloni, M. J.; Kronawitter, C. X.; Runnebaum, R. C. Ethane Dehydrogenation over Cr/ZSM-5: Characterization of Active Sites through Probe Molecule Adsorption FTIR. *Catal. Sci. Technol*. **2020**, *10*(15), 5069-5081. DOI: 10.1039/D0CY01022G

(3) Ravel, B.; Newville, M. ATHENA , ARTEMIS , HEPHAESTUS : Data Analysis for X-Ray Absorption Spectroscopy Using IFEFFIT. *J. Synchrotron Radiat*. **2005**, *12*(4), 537-541. DOI: 10.1107/S0909049505012719

(4) Jain, A.; Ong, S. P.; Hautier, G.; Chen, W.; Richards, W. D.; Dacek, S.; Cholia, S.; Gunter, D.; Skinner, D.; Ceder, G.; Persson, K. A. Commentary: The Materials Project: A Materials Genome Approach to Accelerating Materials Innovation. *APL Mater*. **2013**, *1*(1), 011002. DOI: 10.1063/1.4812323

(5) Kresse, G.; Furthmüller, J. Efficient Iterative Schemes for Ab Initio Total-Energy Calculations Using a Plane-Wave Basis Set. *Phys. Rev. B* **1996**, *54*(16), 11169. DOI: 10.1103/PhysRevB.54.11169

(6) Kresse, G.; Hafner, J. Ab Initio Molecular-Dynamics Simulation of the Liquid-Metal-Amorphous-Semiconductor Transition in Germanium. *Phys. Rev. B* **1994**, *49*(20), 14251. DOI: 10.1103/PhysRevB.49.14251

(7) Kresse, G.; Furthmüller, J. Efficiency of Ab-Initio Total Energy Calculations for Metals and Semiconductors Using a Plane-Wave Basis Set. Comput. *Mater. Sci*. **1996**, *6*(1), 15-50. DOI: 10.1016/0927-0256(96)00008-0

(8) Perdew, J. P.; Burke, K.; Ernzerhof, M. Generalized Gradient Approximation Made Simple. *Phys. Rev. Lett*. **1996**, *77*(18), 3865. DOI: 10.1103/PhysRevLett.77.3865

(9) Hammer, B.; Hansen, L. B.; Nørskov, J. K. Improved Adsorption Energetics within Density-Functional Theory Using Revised Perdew-Burke-Ernzerhof Functionals. *Phys. Rev. B* **1999**, *59*(11), 7413. DOI: 10.1103/PhysRevB.59.7413

(10) Grimme, S.; Ehrlich, S.; Goerigk, L. Effect of the Damping Function in Dispersion Corrected Density Functional Theory. *J. Comput. Chem*. **2011**, *32*(7), 1456-1465. DOI: 10.1002/jcc.21759

(11) Grimme, S.; Antony, J.; Ehrlich, S.; Krieg, H. Consistent and Accurate Ab Initio Parametrization of Density Functional Dispersion Correction (DFT-D) for the 94 Elements H-Pu. *J. Chem. Phys*. **2010**, *132*(15), 154104. DOI: 10.1063/1.3382344

(12) Chen, Y.; Rana, R.; Sours, T.; Vila, F. D.; Cao, S.; Blum, T.; Hong, J.; Hoffman, A. S.; Fang, C. Y.; Huang, Z.; Shang, C.; Wang, C.; Zeng, J.; Chi, M.; Kronawitter, C. X.; Bare, S. R.; Gates, B.C.; Kulkarni, A.R. A Theory-Guided X-Ray Absorption Spectroscopy Approach for Identifying Active Sites in Atomically Dispersed Transition-Metal Catalysts. *J. Am. Chem. Soc*. **2021**, *143*(48), 20144-20156. DOI: 10.1021/jacs.1c07116

(13) Newville, M. Larch: An Analysis Package for XAFS and Related Spectroscopies. *J. Phys. Conf. Ser*. **2013**, *430*, 012007. DOI: 10.1088/1742-6596/430/1/012007
